# Nuclear exosome targeting complexes modulate cohesin binding and enhancer-promoter interactions in 3D

**DOI:** 10.1038/s41467-026-76396-5

**Published:** 2026-08-06

**Authors:** Charbel Akkawi, Alexandre Heurteau, Xavier Contreras, Marion Helsmoortel, Stéphane Schaak, Julie Bossuyt, Olivier Fosseprez, David Dépierre, Olivier Cuvier, Rosemary Kiernan

**Affiliations:** 1 Institut de Génétique Humaine (IGH), Univ Montpellier, CNRS, Montpellier, France; 2 Center of Integrative Biology (CBI), CNRS; Université de, Toulouse, France; 3 Gen2i, Toulouse, France

**Keywords:** Chromatin structure, Long non-coding RNAs

## Abstract

Three-dimensional long-range contacts between enhancers and promoters are thought to be largely determined by loop extrusion driven by the cohesin complex and insulator factors. However, recent evidence also suggests a role for noncoding RNAs, such as enhancer-associated RNAs and promoter upstream transcripts, in shaping enhancer-promoter connectivity. While the nuclear RNA exosome, together with targeting complexes, poly(A) tail exosome targeting connection and nuclear exosome targeting complex, controls the decay of noncoding RNAs, it remains unclear whether these complexes regulate three-dimensional chromatin contacts. Chromatin recruitment maps of the nuclear exosome targeting complex subunit ZCCHC8, the poly(A) tail exosome targeting connection subunit ZFC3H1, and the RNA helicase MTR4 in human cells reveal that these factors associate with sites of enhancer–promoter interactions. Depletion of these factors leads to the accumulation of ncRNAs, notably enhancer-associated RNAs and promoter upstream transcripts, and increases cohesin occupancy at these sites. Chromatin conformation capture analysis reveals that MTR4 modulates long-range enhancer-promoter contacts. Upon loss of MTR4, enhancer-promoter contacts increase while intraloop contacts decrease, suggesting that MTR4 facilitates loop extrusion. These data highlight a key interplay between cohesin-mediated enhancer-promoter interactions and the regulation of noncoding RNAs by nuclear RNA exosome targeting complexes that is consistent with a role for RNA in genome folding.

## Abstract (web summary)

Here, the authors show that components of the nuclear RNA exosome pathway, which normally degrade noncoding RNAs, are recruited to sites of enhancer–promoter interaction, revealing a link between RNA turnover and three-dimensional genome organization.

## Introduction

The human genome needs to be highly compacted in order to occupy a nucleus that measures approximately 10 μm in diameter. Compaction must occur in a manner that permits coordinated gene expression. To achieve this, the genome is organized at multiple scales. Chromosomes are sub-divided into distinct active and inactive (A/B, respectively) compartments, and more locally folded into topologically associating domains (TADs) that correspond to regions favoring high frequencies of contacts between distant sites, such as enhancers and their target promoters, that lie within the same TAD. The establishment of TADs and loop structures is thought to be determined by the coordinated action of CCCTC-binding factor (CTCF) and the cohesin complex, a ring-shaped multimeric structure. TADs are thought to be formed via ‘loop extrusion’, a mechanism in which the cohesin ring entraps chromatin and extrudes loops, until blocked at TAD borders by CTCF or other insulating factors^1–8^.

While the organization of the genome in 3D has been implicated in the regulation of gene expression^2,4,8^, it is less clear how gene expression affects genome folding. Recent evidence suggests that RNAPII and its RNA product may indeed influence 3D genome interactions^9–11^. Several subunits of the cohesin complex, as well as CTCF, bind RNA in addition to DNA. Deletion of the RNA-binding domain of CTCF abolished about 50% of chromatin loops in mESCs^12,13^. Notably, TAD boundaries often express ncRNAs, which stabilize cohesin-CTCF interactions or facilitate CTCF recruitment^14,15^. RNA-guided recruitment of CTCF has been shown to increase the efficiency of target search by several fold^16^. RNAPII was recently shown to be required for certain enhancer-promoter contacts and to antagonize loop extrusion^10,11^. Moreover, the recent development of 3D interaction maps of RNA has revealed interactions between enhancer-associated RNAs (eRNAs) and promoter proximal transcripts (PROMPTs) that parallel the functional connectivity of enhancers and promoters^17^. Mechanisms that control the abundance of such RNAs are likely to affect RNA-RNA spatial interactions and, consequently, genome looping and gene expression.

Many ncRNAs, including enhancer-associated RNAs (eRNAs) and promoter proximal transcripts (PROMPTs), are intrinsically unstable and are degraded by the nuclear RNA exosome, a multi-subunit barrel-shaped structure containing a helicase, MTR4 and 3’–5’ exoribonucleases, Dis3 and Exosc10^18^. RNA substrates must first be delivered to the nuclear exosome, which is carried out by two major targeting complexes: the Nuclear Exosome Targeting Complex (NEXT)^19^ and the polyA tail exosome targeting connection (PAXT)^20^. NEXT consists of a zinc-finger protein, ZCCHC8, together with RBM7 and the shared RNA helicase, MTR4. NEXT targets mostly short RNAs that are not polyadenylated. The second targeting complex, PAXT, is comprised of a zinc-finger protein, ZFC3H1, together with RBM26/RBM27, ZC3H3 and PABPN1, and targets polyadenylated RNAs^20,21^. We recently identified an additional PAXT subunit, polyA polymerase gamma (PAPγ), further strengthening the link between PAXT and polyadenylated RNA substrates^22^. RNAs targeted by PAXT and/or NEXT include not only aberrant transcripts, but many RNAs that are associated with gene regulatory regions, such as enhancer RNAs (eRNAs) and promoter-proximal transcripts (PROMPTs), also known as upstream antisense transcripts (uaRNAs)^21^. While nuclear exosome and its targeting complexes have been well characterized, whether the degradation of certain targets, such as eRNAs, but also PROMPTs and ncRNAs, might affect genome organization and gene expression is not clear.

We recently described the genome-wide mapping of PAXT subunits, ZFC3H1, RBM26 and PAPγ^22^. Here, we have identified the genome-wide localization of the ZCCHC8 subunit of NEXT, as well as the shared helicase, MTR4. Chromatin immunoprecipitation and high-throughput sequencing (ChIP-seq) mapping revealed that the NEXT subunit, ZCCHC8 and the PAXT subunit, ZFC3H1, were detected primarily at transcription start sites (TSSs) but also at active enhancers. Consistent with their localization, loss of these factors was associated with stabilization of PROMPTs and eRNAs, as expected. Loss of MTR4 also strongly stabilized ncRNAs including eRNAs and PROMPTs, as described previously^19–21^. However, ChIP-seq mapping revealed that MTR4 was present at active enhancers, but was notably absent at TSSs, suggesting that its interaction with TSSs may be transient. By integrating ChIP-seq, RNA-seq and chromatin conformation capture (Hi-C) data, our data show that MTR4 contacts TSSs via 3D interactions between enhancers and promoters. Hi-C and ChIP-seq data furthermore showed that MTR4 modulates cohesin-dependent 3D contacts. Loss of MTR4 increases enhancer-promoter contacts at the anchor site but antagonizes loop extrusion. These data suggest that nuclear RNA surveillance factors contribute to chromatin loop formation and help shape genome architecture.

## Results

### PAXT, NEXT and MTR4 are recruited to specific sites on chromatin

**Fig. 1:**
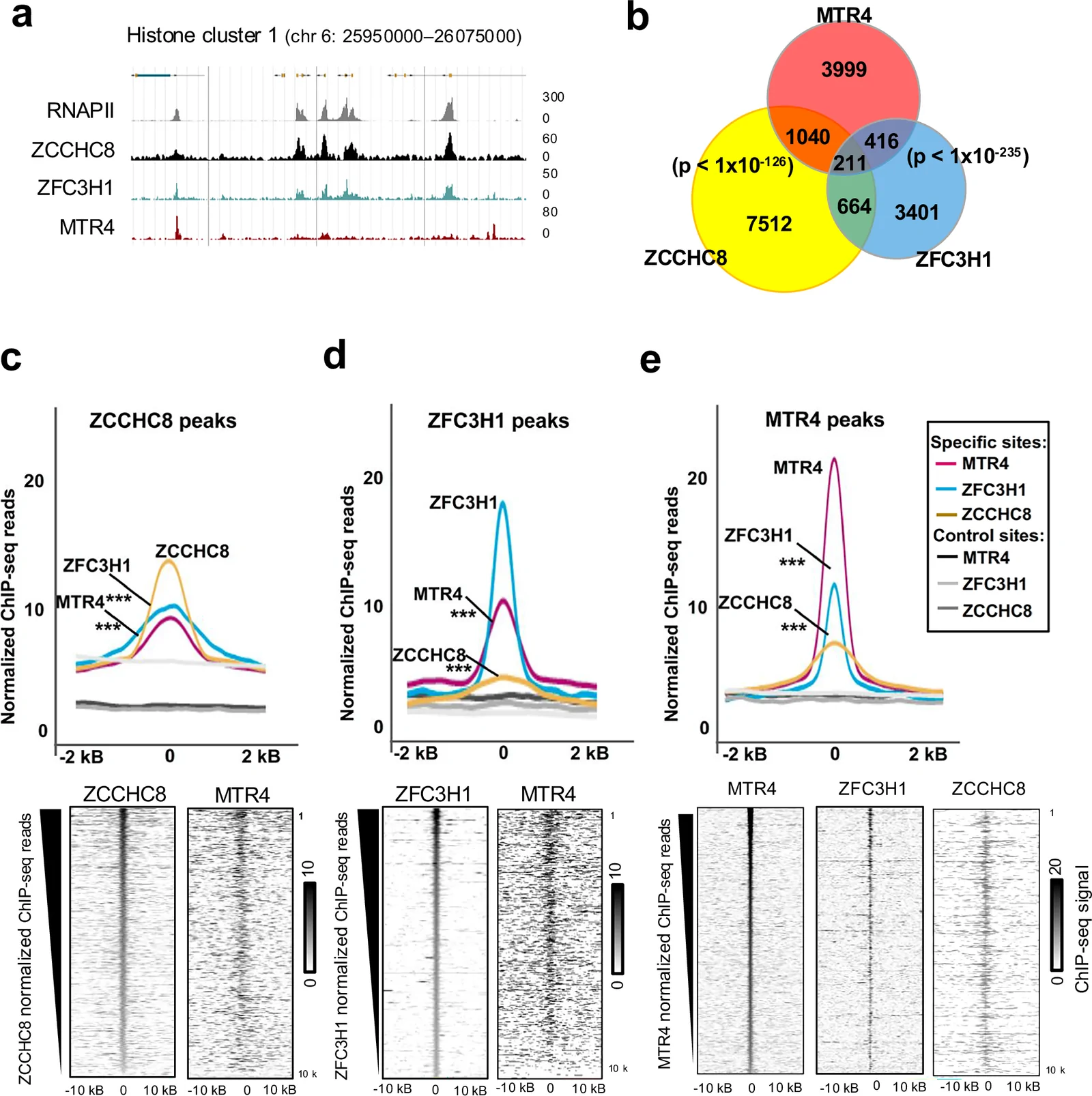
Genome-wide identification of sites bound by MTR4, ZFC3H1 or ZCCHC8. **a** Browsershot showing ChIP-seq reads following precipitation with anti-RNAPII, anti-ZCCHC8, anti-ZFC3H1 or anti-MTR4 specific antibodies, over the indicated region of the human genome. **b** Venn diagram showing the intersection between sites bound by MTR4 and ZFC3H1 (*p* < 1 × 10^−235^) or MTR4 and ZCCHC8 (*p* < 1 × 10^−126^), as calculated by Fisher’s exact test. **c–e** Top Average plots of ChIP-seq reads of ZCCHC8, ZFC3H1 and MTR4, as indicated, at ZFC3H1, ZCCHC8 or MTR4 recruitment sites compared to control sites (see “Methods”). *P*-values were calculated at ZCCHC8, ZFC3H1 or MTR4 specific sites compared to control (random) sites using a one-sided Wilcoxon test of the ZCCHC8 (***, *p* < 1 ×10^−6^), ZFC3H1 (***, *p* < 1 × 10^−6^) or MTR4 (***, *p* < 1 × 10^−6^) ChIP-seq signal as compared to the control signal. bottom Heatmaps centered and rank-ordered on normalized ChIP-seq reads of ZCCHC8, ZFC3H1 and MTR4, as indicated. ChIP-seq reads of ZCCHC8, ZFC3H1 or MTR4 were plotted respecting the same ranking, as indicated.

We have previously shown that PAXT subunits, ZFC3H1, RBM27 and PAPγ, are associated with chromatin, frequently at TSSs that generate PROMPTs^22^. To gain further insight into how the nuclear exosome and its targeting complexes regulate coding and non-coding RNAs through recruitment to sites of transcription, we mapped the genomic localization of NEXT and the shared helicase, MTR4, by chromatin immunoprecipitation combined with high-throughput sequencing (ChIP-seq). While MTR4, ZFC3H1 and ZCCHC8 recruitment sites frequently co-localized, they also showed some notable differences (Fig. 1a and Supplementary Fig. 1). Only a partial overlap between ZFC3H1 and ZCCHC8 recruitment sites was expected, which is consistent with their function in targeting specific RNAs^19–21^ (Fig. 1b–d and Supplementary Fig. 1). MTR4 recruitment sites overlapped with 1251 and 627 recruitment sites of ZCCHC8 and ZFC3H1, respectively (Fig. 1b; Fisher’s exact test: *p* < 1 × 10−^126^ and *p* < 1 × 10−^235^, respectively), consistent with the functions of PAXT or NEXT with MTR4 in targeting specific RNAs^19–21^. Surprisingly, however, the majority of MTR4 sites did not overlap with those of either ZFC3H1 or ZCCHC8, even though MTR4 is a subunit of both PAXT and NEXT macromolecular complexes. Further inspection using averaged profile analysis of ChIP-seq peaks showed that while recruitment of ZCCHC8, ZFC3H1 and MTR4 partially overlapped, ZFC3H1 and MTR4, in particular, showed some specificity in recruitment site preference (Fig. 1c–e, top panels). Ranking of the ChIP-seq signal of ZCCHC8 or ZFC3H1 showed relatively moderate levels of MTR4 (Fig. 1c, d, bottom panels), though significantly above random signal as shown on average profile analysis (Fig. 1c, d, top panels). Similarly, averaged profile analysis and ranking of MTR4 ChIP-seq reads on heat maps further revealed only a modest signal of either ZFC3H1 or ZCCHC8 (Fig. 1e and Supplementary Fig. 1c). These results suggest that while binding sites of ZFC3H1, ZCCHC8 and MTR4 overlap to some extent, many sites appeared to be independent.

### Recruitment of PAXT, NEXT and MTR4 at ncRNA target sites

To further investigate binding profiles of NEXT, PAXT and MTR4, we compared sites of recruitment to those of synthesis of their ncRNA targets. To do so, we generated atlases for coding and non-coding RNA targets, using strand-specific RNA sequencing data obtained from cells depleted of MTR4 (Supplementary Fig. 2a), ZFC3H1 subunit of PAXT and ZCCHC8 subunit of NEXT^20,23,24^. As expected, depletion of MTR4, ZCCHC8 or ZFC3H1 led to the accumulation of thousands of ncRNAs (Supplementary Fig. 2b), using a high confidence threshold for detection (FDR *p* < 1 × 10^−^^6^). The ncRNAs detected in each depletion condition showed significant overlap, with more than 500 ncRNAs detected upon depletion of either ZFC3H1 or ZCCHC8 that were also detected upon MTR4 depletion (Supplementary Fig. 2b; *p* < 1 × 10^−^^102^ and *p* < 1 × 10^−^^149^, respectively) and levels of >200 ncRNAs were increased upon depletion of ZFC3H1, ZCCH8 or MTR4 (Supplementary Fig. 2b; *p* < 1 × 10^−^^121^).

The ncRNAs detected were classified into categories based on their localization with respect to genes (Supplementary Fig. 2c). A significant fraction of ncRNAs detected occur immediately upstream (<2 kb) from transcription start sites (TSSs) of coding genes (Supplementary Fig. 2c, d), corresponding to PROMPTs, in agreement with previous reports^20,21,23^. Approximately 30% of the ncRNAs increased upon depletion ZFC3H1, MTR4 or ZCCHC8 were identified as PROMPTs (Supplementary Fig. 2c). Other categories of ncRNAs increased upon depletion of ZFC3H1, ZCCHC8 or MTR4 include eRNAs, antisense RNAs within gene bodies, and RNAs in intergenic regions (Supplementary Fig. 2c). Among PROMPTs, 62.5% of ZFC3H1-dependent PROMPTs and 51.6% of ZCCHC8-dependent PROMPTs were also increased following MTR4 depletion (Supplementary Fig. 2e). Similarly, 54.6% of ZFC3H1-dependent eRNAs and 42.5% of ZCCHC8-dependent eRNAs were also increased following MTR4 depletion **(**Supplementary Fig. 2f**)**. Our data are thus similar to previous analyses showing the contribution of MTR4-containing complexes in regulating ncRNAs including eRNAs and PROMPTs^19–21^.

**Fig. 2:**
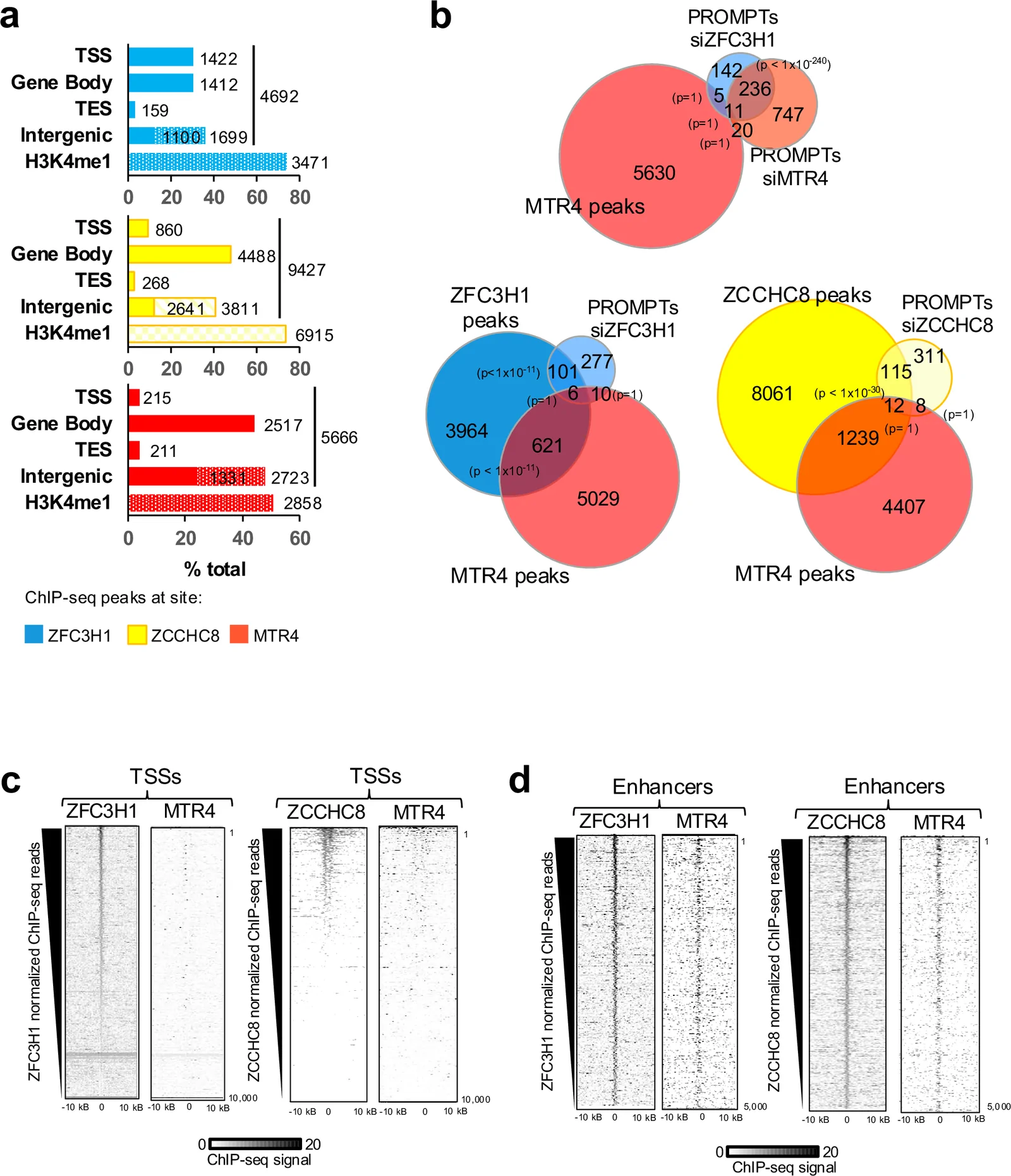
Recruitment sites of MTR4 overlap with those of ZFC3H1 and ZCCHC8 but not with PROMPTs. **a** Histograms showing the genomic distribution of ChIP-seq peaks of ZFC3H1, ZCCHC8 and MTR4, as indicated, overlapping with transcription start sites (TSS), transcription end sites (TES), gene bodies or intergenic (not surrounding gene units ± 2 kb) regions. Peaks overlapping with H3K4Me1 (H3K4me1, all localizations) are indicated as hatched bars; intergenic peaks overlapping with H3K4me1 are shown (hatched) as a proportion of all intergenic peaks. **b** Venn diagrams showing the intersection between PROMPTs detected in cells depleted of MTR4 with the corresponding binding sites of MTR4 (*p* = 1), or ZFC3H1 depletion with ZFC3H1 sites (*p* = 1 × 10^−11^), or ZCCHC8 depletion with ZCCHC8 peaks (*p* = 1 × 10^−30^), as indicated, together with the intersection of PROMPTs detected in cells depleted of MTR4 and ZFC3H1 (*p* = 1 × 10^−240^), MTR4 and ZFC3H1 sites (*p* = 1 × 10^−11^) or MTR4 and ZCCHC8 sites (*p* = 1), as indicated (*P*-values were obtained using Fisher’s exact test). **c** ChIP-seq heatmaps centered and rank-ordered by ZFC3H1 (left) or ZCCHC8 (right) signal at TSSs (± 10 kb). ChIP-seq reads of MTR4 were plotted respecting the same ranking, as indicated. **d** ChIP-seq heatmaps centered and rank-ordered by ZFC3H1 (left) or ZCCHC8 (right) signal at distant enhancer sites (± 10 kb). ChIP-seq reads of MTR4 were plotted respecting the same ranking, as indicated.

We then analyzed the genomic distribution of MTR4, ZFC3H1 and ZCCHC8 ChIP-seq reads (Fig. 2a and Supplementary Fig. 2g). Surprisingly, less than 2% of MTR4 sites localized near a TSS, in contrast to ZFC3H1 sites that were more frequently associated with a TSS, and to a lesser extent ZCCHC8 sites (Fig. 2a). Accordingly, MTR4 recruitment sites accounted for <5% of the PROMPTs detected upon the depletion of ZFC3H1, ZCCHC8, or MTR4 itself (Fig. 2b; *p* = 1). In contrast, ZFC3H1 was detected at 27% of the PROMPTs detected upon its depletion (Fig. 2b; bottom left Venn diagram, *p* < 1 × 10^−^^11^). Similarly, 28% of ZCCHC8-dependent PROMPTS co-localized with a ZCCHC8 recruitment site (Fig. 2b; bottom right Venn diagram).

Consistent with the poor overlap between MTR4 binding sites and PROMPTs, recruitment of MTR4 was essentially not detected at TSSs bound by either ZFC3H1 or ZCCHC8 (Fig. 2c). Correspondingly, the histone mark H3K4me3, typical of active TSSs, was enriched at ZFC3H1 and ZCCHC8 sites but not at MTR4 sites (Supplementary Fig. 2h). In contrast, H3K4me1, characteristic of enhancers, was enriched at sites bound by all three factors, including MTR4 (Fig. 2a and Supplementary Fig. 2g, h). In support of this finding, ZFC3H1- and ZCCHC8-bound enhancers were associated with MTR4 (Fig. 2d). In summary, MTR4 co-localizes with ZFC3H1 or ZCCHC8 primarily at sites that bear marks of active enhancers, in stark contrast with TSSs, where MTR4 recruitment is poorly detected.

### Distal MTR4 sites establish long-range interactions with TSSs that generate PROMPTs

To understand how MTR4 may influence the accumulation of TSS-associated PROMPTs despite being poorly detectable at sites of PROMPT synthesis, we first determined the expression level of flanking genes in the presence and absence of MTR4, reasoning that increased expression of the flanking gene might explain the increase in PROMPTs. While the expression of genes flanking PROMPTs was sometimes deregulated in MTR4-depleted cells (126 genes) (Supplementary Fig. 3a), MTR4-dependent PROMPTs were associated with both up-regulated and down-regulated genes, similarly to ZFC3H1- or ZCCHC8-dependent PROMPTs (Supplementary Fig. 3b–e).

To further exclude the possibility that PROMPT accumulation reflects global transcriptional deregulation, we analysed RNAPII occupancy by ChIP–seq in control and MTR4-depleted cells. Genome-wide RNAPII profiles were highly similar between conditions, and no significant differences were detected at TSSs that accumulate PROMPTs (Supplementary Fig. 3f). These data indicate that MTR4 depletion does not broadly perturb transcription or RNAPII recruitment at promoters, supporting a post-transcriptional mechanism for PROMPT stabilization.

Taken together, these results argue against the possibility that the accumulation of MTR4-dependent PROMPTs was linked to increased expression of the flanking gene. Moreover, these results suggest that the differential expression of genes does not explain the effect of MTR4 on TSS-associated PROMPTs.

**Fig. 3:**
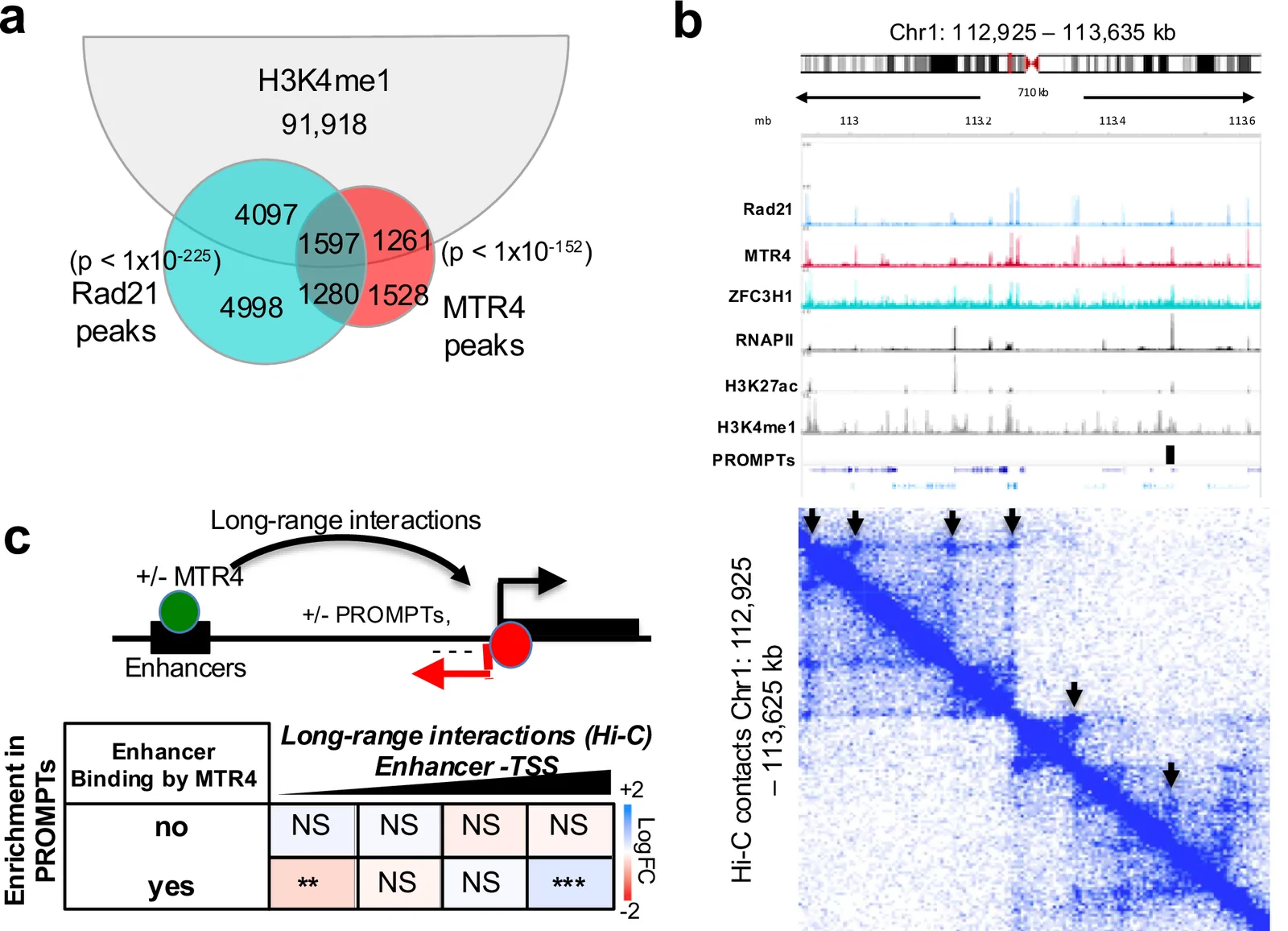
MTR4 influences PROMPTs through long-range interactions. **a** Venn diagram showing the intersection between binding sites of MTR4, Rad21 and the enhancer-associated H3K4me1 histone mark. *P*-values for the intersection between the sites of H3K4me1 and MTR4 (*p* < 1 × 10^−152^) or between H3K4me1 and Rad21 (*p* < 1 × 10^−2^^25^) were obtained using Fisher’s exact test. **b** Top: Genomic view of the binding profiles of RNAPII, MTR4, ZFC3H1 and Rad21. Bottom: 2D matrix representing the levels of normalized long-range interactions within the region, determined from Hi-C data. **c** Top: Schematic representation of the enrichment test performed for enhancer-promoter LRIs, determined by systematic genome-wide interrogation of TSSs from Hi-C data, with the indicated features. Bottom: Matrices showing enrichment for the presence of PROMPTs, depending on levels of LRIs (ordered in quartiles) and co-localization of MTR4 with distant enhancers. TSSs with PROMPTs are enriched in MTR4-bound enhancers, establishing the highest interactions (***, *p* < 1 × 10^−^^6^) and significantly under-represented in those with low LRIs (**, *p* < 1 × 10^−15^) as tested using a Wilcoxon one-sided test.

As MTR4 was recruited to distant sites marked by H3K4me1 (Supplementary Fig. 2h), our data raised an alternative possibility that MTR4, together with ZFC3H1 or ZCCHC8, may be involved in regulating PROMPTs depending on enhancer-promoter long-range interactions. Cohesin preferentially binds to H3K4me1-associated enhancers^25^ to mediate long-range interactions (LRIs) with distant TSSs that may be more or less transient^7,26^. Remarkably, more than half of all MTR4 binding sites co-localized with the Rad21 subunit of cohesin, and a significant proportion co-localized with both Rad21 and H3K4me1 (Fig. 3a and Supplementary Fig. 4a, b; Fisher's exact test *p* < 1 × 10^−^^225^ and *p* < 1 × 10^*−*^^152^).

Assessment of long-range chromatin interactions by Hi-C using 2D interaction maps revealed that many PAXT/NEXT/MTR4 recruitment sites form long-range interactions with PROMPT-associated TSSs (Fig. 3b). To further test a possible role of LRIs in juxtaposing MTR4 to its TSS-associated target, we first performed a genome-wide ranking of all pairs of enhancers/active TSSs (within a 1Mbp window around enhancers) from the highest to lowest interaction, based on normalized levels of Hi-C counts^27–29^ (Fig. 3c and Supplementary Fig. 3g). TSSs were further scored for the presence or absence of PROMPTs. Of interest, PROMPTs were largely enriched at TSSs scoring high interaction levels with distal enhancers bound by MTR4 (Fig. 3c, bottom row of matrix). In contrast, active TSSs having the least contact with distal MTR4 sites were significantly depleted of PROMPTs (Fig. 3c, bottom row). Moreover, TSSs with high levels of contacts with enhancers not bound by MTR4 were not significantly associated with PROMPTs (Fig. 3c, top row), indicating that stronger enhancer-promoter interactions alone do not explain the enrichment of PROMPTs.

The differential levels of LRIs were validated by assessing the averaged physical contacts by aggregation of Hi-C data (aggregated peak analysis, APA), which readily estimates genome-wide LRIs^28,29^. APA showed that LRIs between enhancer/TSSs were clearly higher for the upper quartile, which are enriched in MTR4-bound enhancers and PROMPTs compared to the lower quartile, which is depleted of PROMPTs (Fig. 3d). These data suggest that PROMPTs are enriched at promoters engaged in high levels of LRIs with MTR4-bound enhancers. These data suggest that nuclear exosome targeting complexes bound to distant enhancers are engaged in physical contacts with their target TSSs harboring PROMPTs (Fig. 3c, schematic, top panel). As such, these data raise the possibility that regulation of ncRNAs may involve the juxtaposition of enhancer-bound MTR4 complexes to distal TSSs, in 3D.

### MTR4 is associated with sites of cohesin-dependent long-range interactions and modulates cohesin binding

**Fig. 4:**
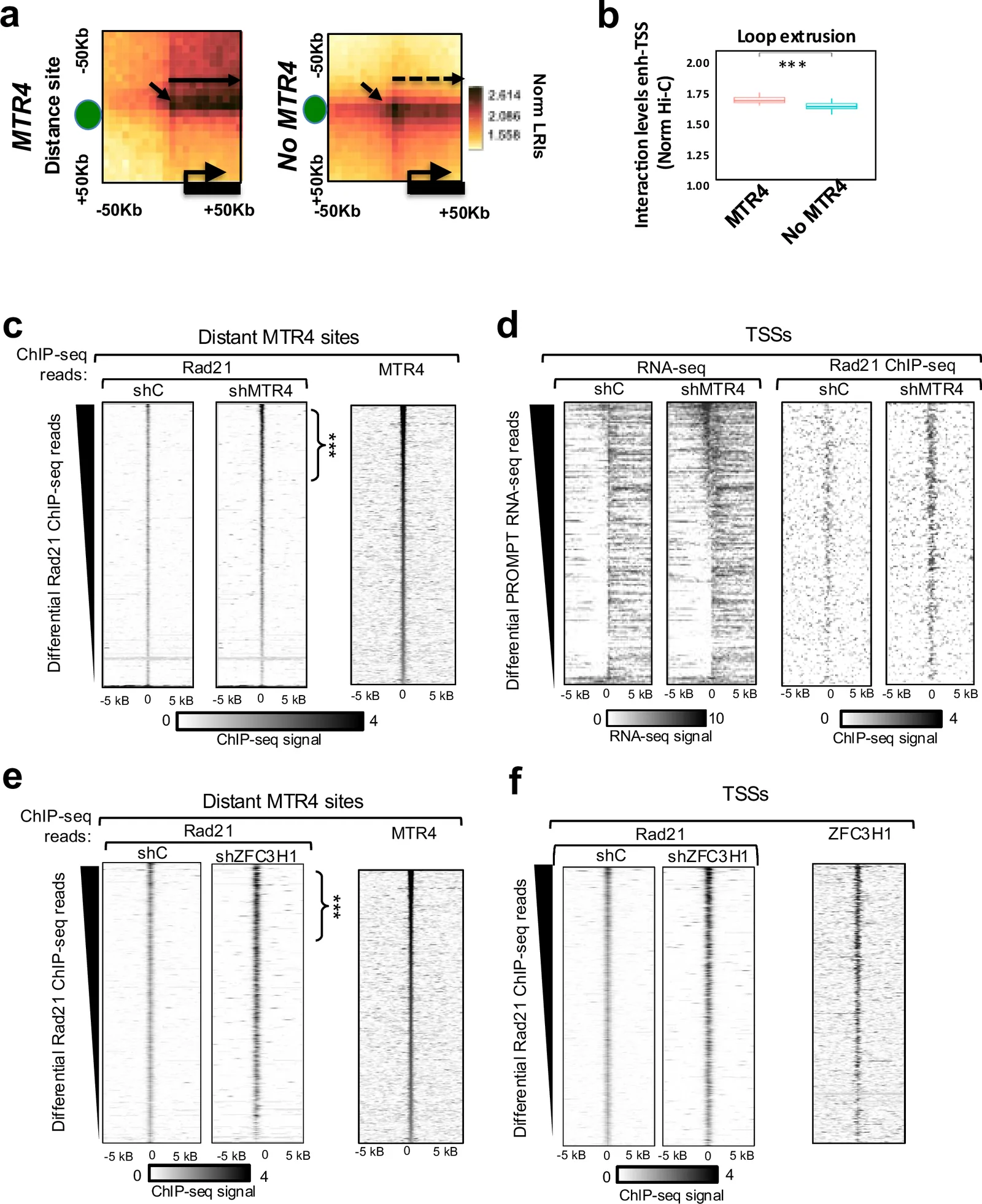
MTR4 is associated with sites of cohesin-dependent long-range interactions and modulates cohesin binding. **a** 2D APA plot representing normalized Hi-C counts at enhancers bound by MTR4 or not (left and right plot, respectively). Aggregation analysis was performed between enhancers and all active TSSs within 1 Mbp. **b** Box plot showing the distribution of long-range interactions along the bodies of genes contacted by distant enhancers, depending on the presence or absence of MTR4. Box plots display the median, upper, and lower quartiles; the whiskers show a 1.5 × interquartile range (***p < 1 × 10^−^^6^, Wilcoxon one-sided test) from independent biological replicates. **c** Heatmaps centered on MTR4 binding sites ± 5 kB, and rank ordered on differential Rad21 ChIP-seq reads in cells depleted of MTR4 (shMTR4) compared to control cells (shC). The bracket represents the most significant differential binding as quantified by z-scores (see “Methods”) for which the significant *p*-value obtained by the Wilcoxon pairwise (two-sided) test was obtained (***, *p* < 1 × 10^−^^6^). MTR4 ChIP-seq reads were plotted respecting the same ranking (right). **d** Heatmaps rank-ordered by increased PROMPTs detected by RNA-seq upon depletion of MTR4 compared to controls, and centered on TSSs ± 5 kB (left). Rad21 ChIP-seq reads were plotted respecting the same ranking. **e** Heatmaps centered on distant MTR4 binding sites ± 5 kB, and rank ordered on differential Rad21 ChIP-seq reads in cells depleted of ZFC3H1 (shZFC3H1) compared to control cells (shC) (left). MTR4 ChIP-seq reads were plotted respecting the same ranking (right). The bracket represents the most significant differential binding as quantified by z-scores for which the significant *p*-value obtained by the Wilcoxon pairwise (two-sided) test was obtained (***, *p* < 1 × 10^−^^6^). **f** Heatmaps of Rad21 upon depletion of ZFC3H1 compared to control, and centered on TSSs ± 5 kB (left). ZFC3H1 ChIP-seq reads were plotted respecting the same ranking (variation in Rad21 binding).

Since MTR4 co-localized significantly with the Rad21 subunit of cohesin and the H3K4Me1 mark associated with active enhancers (Fig. 3a and Supplementary Fig. 4a, b), we tested whether MTR4 might be recruited to cohesin-associated LRIs genome-wide. Physical contacts were probed through aggregation of Hi-C data (APA). LRIs were further oriented in the direction of sense transcription, which allows an estimate of loop extrusion associated with the enhancer-promoter contacts. Hi-C data and MTR4 ChIP-seq data from HeLa cells were analyzed to determine the frequency of cohesin-associated LRIs and loop extrusion, associated with MTR4 co-localization or not (Fig. 4a). Firstly, the analysis revealed that MTR4 is present at distal enhancers that are engaged in 3D interactions with TSSs and that these LRIs are associated with loop extrusion (Fig. 4a, left panel). Although this phenomenon could be general, we noted that LRIs involving enhancers that were not associated with MTR4 (No MTR4) showed significantly less loop extrusion (Fig. 4a; right panel, Fig. 4b). This is likely to be due at least in part to low Rad21 association at these sites, since MTR4 recruitment sites frequently co-localize with Rad21 (Fig. 3a and Supplementary Fig. 4a, b). Indeed, LRIs between Rad21-associated enhancers and TSSs showed strong loop extrusion while enhancers that were not associated with Rad21 showed significantly lower levels of loop extrusion, as expected (Supplementary Fig. 4c, d). Thus, MTR4 is associated with enhancers that engage in LRIs with TSSs and form extruded genome loops.

We next asked whether MTR4 influences cohesin binding. ChIP–seq for Rad21 in MTR4-depleted cells revealed increased Rad21 binding at several hundred MTR4 sites (Fig. 4c and Supplementary Fig. 5a, b). Of note, Rad21 protein levels were unchanged in extracts of MTR4-depleted cells compared to controls (Supplementary Fig. 5c), indicating that the observed accumulation was not due to increased Rad21 expression. To determine whether such increases might be related to the abundance of ncRNAs, TSSs were then ranked by the accumulation of PROMPTs following MTR4 depletion (Fig. 4d, left heatmaps, highest to lowest PROMPT accumulation). Accumulation of PROMPTs at TSSs correlated strikingly with gain in Rad21 ChIP-seq signal in MTR4-depletion condition (Fig. 4d, right heatmaps). Furthermore, TSSs at which Rad21 accumulated most upon MTR4 depletion were also those at which PROMPTs increased upon depletion of ZFC3H1 or ZCCHC8, in addition to MTR4 (Supplementary Fig. 5d). We also observed that the most significant effect was associated with the differential expression of the flanking genes (Supplementary Fig. 5e), suggesting an association with gene expression. In addition, repeating the same analysis upon depletion of ZFC3H1 (Supplementary Fig. 6a) confirmed the impact on Rad21 signal (Fig. 4e, f), indicating that cohesin association is modulated by MTR4, likely in the context of PAXT. Accumulation of cohesin was similarly found over distant MTR4 sites and TSSs, depending on ZFC3H1 depletion. As observed for MTR4, depletion of ZFC3H1 did not alter the expression of Rad21 in cell extracts (Supplementary Fig. 6b). Therefore, our results show that nuclear RNA exosome targeting complexes, which regulate the abundance of non-coding RNAs, also modulate the accumulation of cohesin at the same sites.

**Fig. 5:**
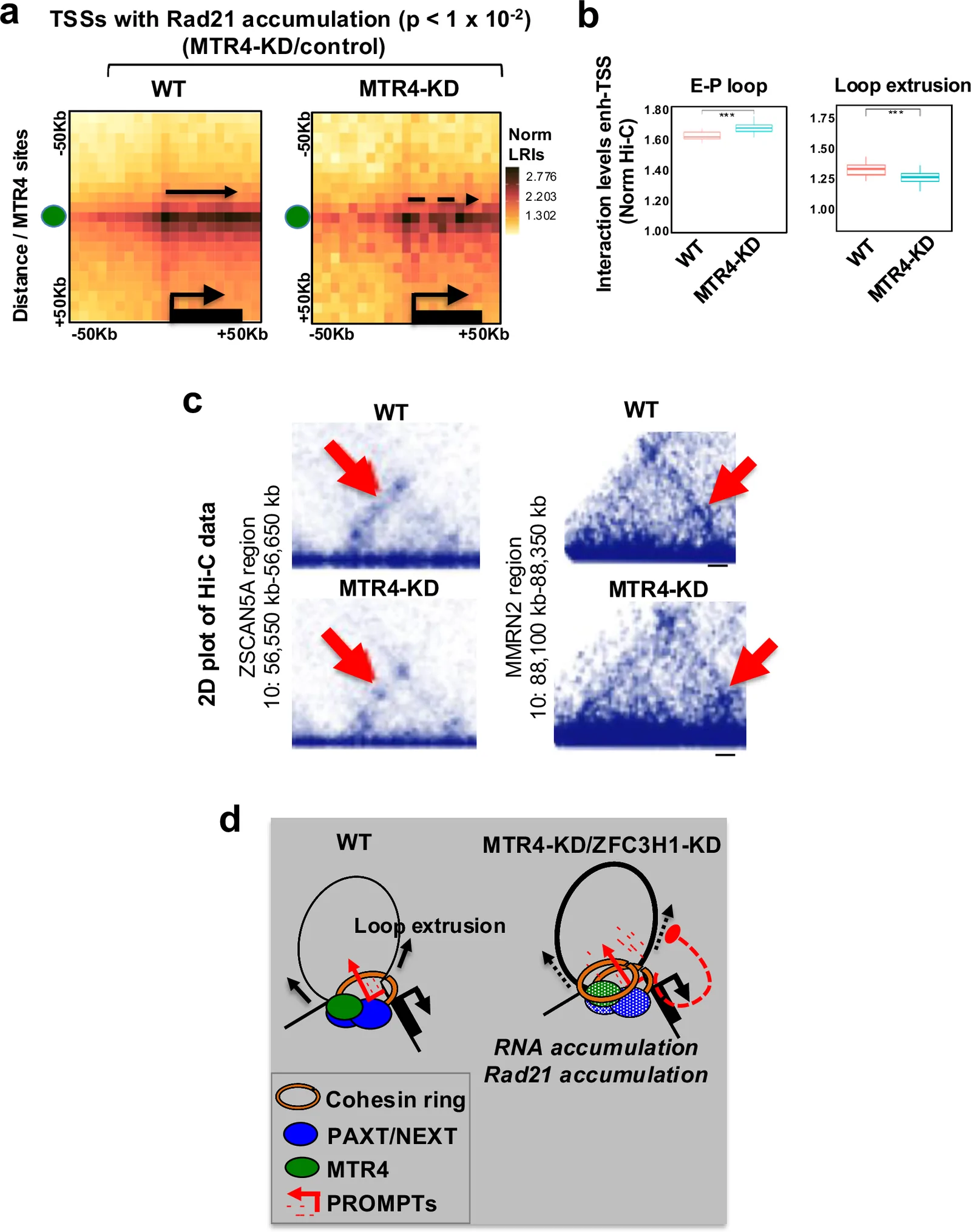
Nuclear exosome targeting complexes influence cohesin-mediated long-range interactions. **a** 2D APA plots representing normalized Hi-C counts at distant MTR4 sites in control or MTR4-depleted conditions (left and right plot, respectively). Aggregation analysis was performed between enhancers and all active TSSs within 1 Mbp. **b** Box plot showing the distribution of long-range interactions at interaction sites (E-P loop, left box plot) or along the bodies of genes contacted by distant enhancers (loop extrusion, right box plot) in control or MTR4-depleted conditions, as indicated. Box plots display the median, upper, and lower quartiles; the whiskers show a 1.5 × interquartile range (***p < 1 × 10^−^^6^, Wilcoxon pairwise (two-sided) test) from independent biological replicates. **c** Browser shots of Hi-C signal over representative regions in control and MTR4 knock-down cells, as indicated. Genomic coordinates in GRCh37 are shown on the left. **d** A schematic representing a model where cohesin-mediated looping is influenced by PAXT, NEXT and MTR4. In wild-type cells (WT), ncRNAs, including PROMPTs and eRNAs, are degraded by nuclear exosome targeting complexes found at loop contact sites. MTR4-depleted cells (MTR4-KD) show accumulation of eRNAs and PROMPTs and stabilization of cohesin-mediated loops (thick black line). Note that eRNAs may still be degraded at enhancers since MTR4 binds to enhancers with PAXT/NEXT, independently of cohesin-mediated looping, in contrast to MTR4-mediated degradation of PROMPTs at promoters that is associated with chromatin looping.

Since the accumulation of cohesin, which is involved in the formation of long-range contacts, was modulated by MTR4 at its recruitment sites, we wondered whether its depletion might also affect 3D genome interactions. Thus, aggregation of Hi-C data obtained from control cells and cells depleted of MTR4 was performed (Fig. 5a). TSSs showing accumulation of Rad21 following loss of MTR4 (*p* < 10^−2^) were selected for analysis. Consistent with an increased association of Rad21, LRIs between TSSs and distant sites were significantly enhanced at the E–P loop (Fig. 5a, b, left panels). Of interest, contacts in the intra-loop region (loop extrusion) were significantly decreased following loss of MTR4 compared to the control condition (Fig. 5a, b, right panels). Interestingly, this pattern resembles that of LRI sites that are not associated with MTR4 in control conditions (Fig. 4a, b). Changes in LRIs were visible at specific MTR4-associated genomic regions (Fig. 5c).

To further investigate whether these changes are linked to MTR4-dependent ncRNA stabilization, we stratified enhancer-promoter contacts based on MTR4 occupancy in control cells and ncRNA accumulation following MTR4 depletion. This analysis revealed that enhancer–promoter interactions increased most prominently at sites that are bound by MTR4 under control conditions and that accumulate PROMPTs (left panel) or eRNAs (right panel) upon MTR4 depletion (Supplementary Fig. 6c). These results support the notion that MTR4-dependent stabilization of ncRNAs contributes to the modulation of long-range chromatin interactions.

Finally, to determine whether depletion of ZFC3H1 also affected long-range chromatin interactions, consistent with its effect on increasing Rad21 accumulation, Hi-C profiles from cells depleted of ZFC3H1 and control cells were analyzed. Significant alterations in long-range interactions were observed, similar to those detected upon MTR4 depletion (Supplementary Fig. 6d, e). This supports the conclusion that nuclear exosome targeting complexes, rather than MTR4 alone, modulate enhancer–promoter contacts.

Taken together, these data show that LRIs are influenced by nuclear RNA exosome targeting complexes containing MTR4.

## Discussion

Here, we identify an intricate regulation of 3D long-range interactions between enhancers and TSSs by nuclear RNA exosome targeting complexes. We began by mapping the genomic localization of targeting complexes, PAXT and NEXT, via ZFC3H1 and ZCCHC8 subunits, respectively, and their shared RNA helicase subunit, MTR4. Comparison between the localizations of the complexes and ncRNA accumulated upon their depletions revealed an interplay between the stability of ncRNAs, including PROMPTs and eRNAs, and 3D long-range interactions. Nuclear exosome targeting subunits were found to be localized at sites that engage in long-range genomic interactions. Moreover, loss of nuclear exosome targeting subunits, MTR4 or ZFC3H1, led to significant changes in the accumulation of cohesin, a key player in long-range interactions, at both TSSs and enhancers and modified the dynamics of chromatin looping (Fig. 5d).

Increasing evidence suggests that RNAPII and its RNA product may play a role in genome organization. Treatment with RNase abolished almost half of all genome contacts^12^. CTCF and cohesin subunits are RNA-binding proteins, and mutation of the RNA-binding region (RBR) of CTCF significantly disrupted CTCF-dependent insulation of chromatin domains and loop extrusion^12,13^. More recently, RNAPII has been shown to shape genome architecture by blocking loop extrusion while facilitating enhancer-promoter contacts^10,11^.

Consistent with these observations, our data show that depletion of nuclear RNA exosome targeting complexes impacts 3D genome architecture and binding of cohesin. It seems unlikely that MTR4 or associated complexes, such as PAXT, are directly responsible for the effects observed on LRIs. Rather, we propose that ncRNA targets of nuclear exosome targeting complexes, particularly eRNAs and PROMPTs, that become stabilized at interacting enhancers and promoters, contribute to altered LRIs and loop extrusion. This model is supported by recent studies demonstrating that enhancer–promoter interactions are facilitated by RNAPII10^10,11^ and by pairwise interactions between eRNAs and PROMPTs^17^. Thus, stabilization of ncRNAs following depletion of nuclear exosome targeting complexes would be expected to modify RNA–RNA spatial interactions and, consequently, genome looping and gene expression.

At the same time, we cannot exclude an alternative, non-mutually exclusive mechanism in which increased Rad21 occupancy reflects changes in chromatin accessibility or regulatory element availability upon depletion of nuclear exosome targeting complexes. Stabilization of ncRNAs at promoters and enhancers may influence local chromatin states, for example, through effects on nucleosome positioning, RNAPII residence, or retention of transcription-associated factors, thereby creating a chromatin environment more permissive for cohesin loading or retention^30^. In this scenario, increased cohesin binding would arise indirectly from altered chromatin architecture rather than from a direct stabilizing effect of ncRNAs on cohesin itself. Discriminating between these mechanisms will require future experiments directly interrogating chromatin accessibility and cohesin dynamics at high temporal resolution.

Interestingly, although interactions between enhancers and TSSs and association with the Rad21 subunit of cohesin were increased, loop extrusion appeared to be less efficient. We speculate that insulator proteins, such as CTCF, Mediator^31^ or RNA-binding proteins, such as hnRNPK^17^, may be retained at the LRI sites, which may impede or contribute to a reduction in loop extrusion. The genome-wide mapping of nuclear exosome targeting complexes, described previously^22^ and in the present study, reveals a strong association with TSS regions of actively transcribing genes and RNAPII. While still speculative, these findings raise the possibility that an important function of these complexes may be to fine-tune the abundance of TSS-associated and enhancer-associated RNAs and, consequently, modulate genome architecture.

An unexpected finding of genome-wide mapping was the absence of detectable MTR4 at TSSs, even though subunits of PAXT and NEXT are readily detectable at TSSs. MTR4 was more stably associated with enhancer regions and was present at sites engaged in long-range interactions with TSSs. This might suggest that cohesin-mediated LRIs may influence ncRNAs post-transcriptionally, depending on the spatial targeting of the nuclear RNA exosome targeting complexes. Interestingly, Rahman et al. demonstrated that eRNA synthesis precedes looping and that their production is strongly reduced in the closed loop conformation^32^. Thus, chromatin loops might help degrade eRNAs when they are no longer required, by increasing recruitment of nuclear exosome targeting complexes, or facilitating the activation of nuclear exosome locally, by juxtaposing the targeting complexes with MTR4 helicase, whose binding is required to activate nuclear exosome. LRIs may thus define the context in which nuclear exosome and its targeting complexes degrade ncRNAs, notably eRNAs and antisense PROMPTs. Improper accumulation of these ncRNAs may deregulate, either directly or indirectly, the expression of the neighboring genes.

## Methods

### Cell culture and reagents

HeLa cells (ATCC, CCL-2) were grown in Dulbecco’s modified Eagle’s minimal essential medium (DMEM) (Sigma-Aldrich, D6429), supplemented with 10% fetal calf serum (FCS; Eurobio Scientific, CVFSVF00-01) and containing 1% penicillin-streptomycin (Sigma-Aldrich, P4333). Cells were grown in a humidified incubator at 37 °C with 5% CO2.

### Antibodies

Antibodies used in this study are listed in Supplementary Table 1. For chromatin immunoprecipitation followed by sequencing (ChIP–seq), 4 μg of antibody were used per experiment. The following antibodies were employed: anti-MTR4 (Abcam, ab70552), anti-ZFC3H1 (Bethyl Laboratories, A301-614), anti-ZCCHC8 (Bethyl Laboratories, A301-805A), anti-RNA polymerase II (clone F12, Santa Cruz Biotechnology, sc-55492), and anti-Rad21 (Abcam, ab992).

For western blot analyses, the following antibodies were used: anti-MTR4 (Bethyl Laboratories, A301-614A, 1:1000), anti-ZFC3H1 (Bethyl Laboratories, A301-456A, 1:1000), anti-ZCCHC8 (Bethyl Laboratories, A301-806A, 1:1000), anti-Rad21 (Abcam, ab992, 1:1000), and anti-tubulin (Sigma-Aldrich, T9026, 1:10,000).

### RNAi

Production of short hairpin RNA (shRNA)-expressing lentiviral particles was performed using plasmids expressing shRNAs targeting MTR4 (Sigma-Aldrich MISSION shRNA, TRCN0000296301), ZFC3H1 (Sigma-Aldrich MISSION shRNA, TRCN0000130498) or a non-targeting control (Addgene, plasmid 1864). For knockdown experiments, HeLa cells were either transduced with lentiviral particles and harvested 5 days later, or transfected with siRNAs (10 nM final concentration, purchased from Eurofins or IDT), shown in Supplementary Table 2 using Interferin (PolyPlus) and harvested 72 h later^33^. RNAi depletions were validated by immunoblot of total cell extracts.

### Sample preparation for RNA-seq, ChIP-seq and Hi-C

For RNA-seq, total RNA was extracted from HeLa cells using TRIzol (Thermo Fisher Scientific) according to the manufacturer’s instructions. RNA-seq (paired end, 125 bp) was carried out by BGI Genomic Services in triplicate.

Chromatin immunoprecipitation followed by high-throughput sequencing (ChIP-seq) was performed from HeLa cells using the ChIP-IT High Sensitivity® kit from Active Motif (ref #53040) according to the manufacturer’s instructions^34^. Each ChIP used 30 μg of chromatin along with 4 μg of antibody detecting MTR4, ZFC3H1, ZCCHC8 or Rad21 (Supplementary Table 1). ChIP-seq libraries were constructed using the Next Gen DNA Library Kit (Active Motif 53216 and 53264). Library quality was assessed using Agilent 2100 Bioanalyzer and Agilent High Sensitivity DNA assay. High-throughput sequencing (PE75) was performed by the Sequencing-By-Synthesis technique using a NextSeq 500 (Illumina) at Genom’ic facility, Institut Cochin, Paris.

Hi-C was performed from HeLa cells using the Arima Hi-C ® kit from Arima Genomics (ref #A510008) according to the manufacturer’s instructions. Libraries were prepared using the KAPA® Hyper Prep kit (KK8500 and KK4824) and Illumina adapters (ref 20016329). High-throughput sequencing (PE150) was performed by Novogene (NovaSeq) or BGI (DNBseq).

### RNA-seq alignment and ChIP-seq data integration

Raw RNA-seq reads were aligned to the hg19 reference genome using the STAR aligner in paired-end, strand-specific mode. The resulting BAM files were then split by strand and used for differential expression analysis.

After quality control using the fastQC tool, ChIP-seq reads were aligned on the hg19 reference genome using Burrows-Wheeler Aligner (BWA, http://bio-bwa.sourceforge.net/)^35^ and then normalized on the corresponding input as described previously^36^. ChIP-Seq peak calling was performed in all conditions (depleted or not) with MACS2 with R 3.4.2 version. Gene TSSs associated with such peaks were identified by overlapping within the +/− 500 bp region around TSSs. Enhancers associated with peaks were identified by overlapping the peak summit with each enhancer range (+/−1000bp). Overlapping analyses were performed using the ‘GenomicRanges’ package functions of R (https://bioconductor.org/packages/ release/bioc/html/ GenomicRanges.html). Venn Diagrams and statistical enrichment tests were performed using Fisher’s exact test or proportional tests. ChIP–seq signal was quantified at the indicated genomic regions, and box plots were generated for the corresponding gene sets. Statistical significance between WT, MTR4-KD, ZFC3H1-KD, and ZCCHC8-KD conditions was assessed using a two-sided Wilcoxon test on biological replicates. Boxplots were generated using ggplot2^37^ R package functions (https://cran.r-project.org/web/packages/ggplot2/index.html). Heatmaps were generated by aligning genes (oriented towards the right) on TSSs (position 0). Genes were ranked according to normalized ChIP-seq reads (TSS +/− 500 bp) of ZFC3H1, followed by visualization of the indicated ChIP-seq reads (of ZFC3H1, MTR4 or ZCCHC8) or of ncRNAs. Visualization of heatmaps was performed with the SeqPlot package^38^. H3K4me1 and Rad21 peaks on HeLa cells were obtained from the Encode Project (GEO accession GSM798322 and GSM935571, respectively).

### Detection of non-coding RNAs

Differential non-coding RNA levels were detected in MTR4 (this study), ZFC3H1 or ZCCHC8 depleted cells^20,23,24^. NcRNAs levels were compared between each condition and with control mock-depleted (‘WT control’) cells using the enrichR function from NormR (binsize of 500 bp, FDR < 0.05)^39^.

### Heatmaps of RNA-seq and ChIP-seq data

Heatmaps were generated by aligning genes (oriented to the right) on TSSs (position 0) and by plotting the normalized bigwig signal on the reverse and forward strand for RNA-seq, simultaneously for each condition. Genes were ranked according to normalized ChIP-seq reads (TSS +/− 500 bp) of a given feature, as indicated in the figure. Visualization of heatmaps was performed with the SeqPlot package^38^.

### Software

All data were processed using R (v3.4.2) and formatted with the GenomicRanges package (v1.30.3). ChIP–seq reads were aligned to the reference genome using the Burrows–Wheeler Aligner (BWA; v0.7.17)^35^. RNA–seq reads were aligned using the STAR aligner (v2.5.4b) in strand-specific mode, followed by filtering of low-count regions using HTSFilter (v1.18.0). Differential expression analysis was performed using DESeq2 (v1.18.1).

ChIP–seq peak calling was carried out with MACS2 (v2.1.1.20160309), and Hi-C data were pre-processed using Juicebox (v1.7). Boxplots, scatterplots, histograms and profile plots were generated in R (v3.4.2) using the ggpubr package (v0.6.0), based on the ggplot2 package (ggplot3.3.2)^37^ as well as SeqPlots (v1.16.0). Venn diagrams were generated using the Vennerable package (v1.0).

### Computational integration of Hi-C data

Hi-C data at 5 Kb resolution were obtained from WT HeLa cells or from cells depleted of MTR4. Datasets were validated by comparison with published Hi-C data from WT HeLa cells (GSE63525)^25^ and from WT and PDS5-depleted cells (GSE102884)^40^.

Long-range contacts were extracted using the ‘dump’ command from Juicer tools to obtain normalized contact matrices (observed/expected and Knight-Ruiz normalization for matrix balancing), which were subsequently filtered to retain interactions within HeLa TADs.

‘Aggregate Peak Analysis’ (APA)^29,41^ plots were generated using the heatmap function from the R stats package after importing matrices in 2D/3D format, as described for APA^29,41^. Analyses were performed on normalized Hi-C matrices at 10 kB resolution, as described^28,29,41^.

Long-range contacts were extracted using the dump command from Juicer tools to obtain normalized contact matrices (observed/expected and Knight–Ruiz normalization for matrix balancing), which were subsequently filtered to retain interactions within HeLa topologically associating domains (TADs). Aggregate peak analysis (APA) plots were generated using the heatmap function from the R stats package after importing the matrices in 2D/3D format, as described for APA^29,41^.

Analyses were performed on normalized Hi-C matrices at 10 kb resolution, as previously described^28,29,41^, with minor adaptations.

A distance threshold of 200 kb to 1 Mb was applied, and each pair of loci was oriented to align interactions between distal enhancers ( ± cohesin/MTR4 binding) and TSSs, with gene bodies oriented to the right of the APA matrix. For statistical analysis, interaction matrices were subsampled 50 times, each containing 5000 locus pairs per condition. Differences between MTR4-depleted and control cells were assessed by comparing the central square values (3 × 3 pixels) of each aggregated matrix using biological Hi-C replicates and a two-sided Wilcoxon test (stat_compare_means, ggpubr v0.6.0).

Loop extrusion was estimated by quantifying signal in pixels located to the right (3′ direction) of the central pixel and comparing these values to loop anchor signals using equivalent numbers of pixels (3 × 3 central square or 9 pixels to the right) across the 50 aggregated matrices. Statistical comparisons were performed using a two-sided Wilcoxon test (stat_compare_means, ggpubr v0.6.0).

For enrichment analysis, enhancer–TSS interactions within 200 kb to 1 Mb were ranked according to normalized Hi-C contact strength and partitioned into quartiles (q1–q4, from low to high interaction levels). The proportion of a given feature (e.g., MTR4 binding sites) was calculated for each quartile, and enrichment was assessed using Fisher’s exact test across all pairwise comparisons.

Visualization of Hi-C signal was performed using Juicebox using default parameters^42^.

### Statistical analyses of ChIP-Seq data

After quality control using fastQC, ChIP-seq reads for MTR4, ZFC3H1, ZCCHC8 and Rad21 were aligned to the hg19 reference genome using BWA. Signal was normalized against the corresponding input control. ChIP-Seq peaks were called using MACS2 with input normalization, generated in R. All subsequent analyses were performed in R (v3.4.2). Genes associated with peaks were identified by overlap within +/− 500 bp of TSSs, as annotated in the UCSC TxdB database (hg19). Enhancers were identified based on by CAGE-seq date from ENCODE. Overlap analyses were performed using functions from the ‘GenomicRanges’ package (https://bioconductor.org/packages/release/bioc/html/GenomicRanges.html), as described previously^36^. Enrichment analyses in Venn diagrams and enrichment matrices were performed using Fisher’s exact test, with one-sided or two-sided tests applied as appropriate to assess enrichment or depletion (Odds ratio >1; alternative = ‘greater’ in R Fisher’s exact test). Proportional Venn diagrams were generated using the ‘Vennerable’ R package (https://github.com/js229/Vennerable).

Strand-specific read quantification was performed by aligning stranded reads over oriented TSSs (+/−5 kb). For analyses of enhancer-promoter associations, enhancers were those defined by CAGE-seq datasets from the Functional annotation of the mammalian genome (FANTOM5) and ENCODE projects^43,44^.

### Statistical analyses of gene expression data

Gene expression analyses were first performed by poly(A) + RNA-seq data from HeLa cells depleted of MTR4 (MTR4-KD) and ZFC3H1 (ZFC3H1-KD), as compared to siRNA-treated control cells (WT). Strand-specific, normalized RNA-seq reads were visualized using the IGV genome browser. Quantification of strand-specific reads was performed in biological duplicates and visualized as heatmaps, with genes ranked according to the indicated ChIP-seq signal levels.

Lowly expressed genes were filtered using the HTSFilter R package, and differential expression analysis was performed using the DESeq2 R package to compare expression between MTR4-KD or ZFC3H1-KD and WT conditions. Differentially expressed genes were defined using a *p*-value threshold <0.05 and a Log2 fold-change threshold of > 1.5 or < −1.5, respectively.

Enrichment between differentially expressed gene sets and other genomic features, including those associated with ChIP-seq peaks, was assessed using Fisher’s exact test. Intersection matrices were generated using ‘GenomicRanges’ functions, with statistical significance evaluated using Fisher’s exact test.

### Reporting summary

Further information on research design is available in the Nature Portfolio Reporting Summary linked to this article.

## Supplementary information

Supplementary Information

Reporting summary

Transparent Peer Review file

## Source data

Source Data

## Data Availability

RNA-seq, ChIP-seq and Hi-C data generated in this study have been deposited at GEO under accession numbers: GSM4263550 (https://www.ncbi.nlm.nih.gov/geo/query/acc.cgi?acc=GSM4263550, ChIP-seq, anti-RNAPII), GSE154100 (https://www.ncbi.nlm.nih.gov/geo/query/acc.cgi?acc=GSE154100, ChIP-seq samples and RNA-seq samples) and GSE283061 (https://www.ncbi.nlm.nih.gov/geo/query/acc.cgi?acc=GSE283061, Hi-C samples). Previously published data analyzed in this study are available at GEO under accession numbers GSE63525 (https://www.ncbi.nlm.nih.gov/geo/query/acc.cgi?acc=GSE63525, Hi-C data) and GSE102884 (https://www.ncbi.nlm.nih.gov/geo/query/acc.cgi?acc=GSE102884, Hi-C data). GSM798322 (https://www.ncbi.nlm.nih.gov/geo/query/acc.cgi?acc=GSM798322, ChIP-seq, H3K4me1, Hela cells) GSM935571 (https://www.ncbi.nlm.nih.gov/geo/query/acc.cgi?acc=GSM935571, ChIP-seq, Rad21 HeLa cells). All data are available in the main text or the supplementary information/Source Data file. Source data are provided with this paper.

## Code availability

Scripts are available at https://github.com/CuvierLab/RNAexosome-3D_interactions-Gen2i.

## References

[1] Rowley, M. J. & Corces, V. G. Organizational principles of 3D genome architecture. *Nat. Rev. Genet*. **19**, 789–800 (2018). 10.1038/s41576-018-0060-8 PMC6312108 30367165

[2] Bonev, B. & Cavalli, G. Organization and function of the 3D genome. *Nat. Rev. Genet.* **17**, 661–678 (2016). 27739532 10.1038/nrg.2016.112

[3] Ea, V., Baudement, M. O., Lesne, A. & Forne, T. Contribution of topological domains and loop formation to 3D chromatin organization. *Genes. (Basel)* **6**, 734–750 (2015). 10.3390/genes6030734 PMC4584327 26226004

[4] Merkenschlager, M. & Nora, E. P. CTCF and cohesin in genome folding and transcriptional gene regulation. *Annu. Rev. Genomics Hum. Genet.* **17**, 17–43 (2016). 27089971 10.1146/annurev-genom-083115-022339

[5] Rowley, M. J. & Corces, V. G. The three-dimensional genome: principles and roles of long-distance interactions. *Curr. Opin. Cell Biol.* **40**, 8–14 (2016). 26852111 10.1016/j.ceb.2016.01.009 PMC4887315

[6] Szabo, Q., Bantignies, F. & Cavalli, G. Principles of genome folding into topologically associating domains. *Sci. Adv.* **5**, eaaw1668 (2019). 30989119 10.1126/sciadv.aaw1668 PMC6457944

[7] Kagey, M. H. et al. Mediator and cohesin connect gene expression and chromatin architecture. *Nature* **467**, 430–435 (2010). 20720539 10.1038/nature09380 PMC2953795

[8] Dekker, J. & Mirny, L. The 3D genome as moderator of chromosomal communication. *Cell* **164**, 1110–1121 (2016). 26967279 10.1016/j.cell.2016.02.007 PMC4788811

[9] Fosseprez, O. & Cuvier, O. Uncovering the functions and mechanisms of regulatory elements-associated non-coding RNAs. *Biochim Biophys. Acta Gene. Regul. Mech.* **1867**, 195059 (2024). 10.1016/j.bbagrm.2024.195059 39226990

[10] Barshad, G. et al. RNA polymerase II dynamics shape enhancer-promoter interactions. *Nat. Genet*. **55**, 1370–1380 (2023). 10.1038/s41588-023-01442-7 PMC10714922 37430091

[11] Zhang, S., Ubelmesser, N., Barbieri, M. & Papantonis, A. Enhancer-promoter contact formation requires RNAPII and antagonizes loop extrusion. *Nat. Genet*. **55**, 832–840 (2023). 10.1038/s41588-023-01364-4 37012454

[12] Hansen, A. S. et al. Distinct classes of chromatin loops revealed by deletion of an RNA-binding region in CTCF. *Mol. Cell* **76**, 395–411 e13 (2019). 31522987 10.1016/j.molcel.2019.07.039 PMC7251926

[13] Saldana-Meyer, R. et al. RNA interactions are essential for CTCF-mediated genome organization. *Mol. Cell* **76**, 412–422 e5 (2019). 31522988 10.1016/j.molcel.2019.08.015 PMC7195841

[14] Isoda, T. et al. Non-coding transcription instructs chromatin folding and compartmentalisation to dictate enhancer-promoter communication and T cell fate. *Cell* **171**, 103–119 e18 (2017). 28938112 10.1016/j.cell.2017.09.001 PMC5621651

[15] Luo, H. et al. HOTTIP-dependent R-loop formation regulates CTCF boundary activity and TAD integrity in leukemia. *Mol. Cell* **82**, 833–851 e11 (2022). 35180428 10.1016/j.molcel.2022.01.014 PMC8985430

[16] Hansen, A. S., Amitai, A., Cattoglio, C., Tjian, R. & Darzacq, X. Guided nuclear exploration increases CTCF target search efficiency. *Nat. Chem. Biol.* **16**, 257–266 (2020). 31792445 10.1038/s41589-019-0422-3 PMC7036004

[17] Cai, Z. et al. RIC-seq for global in situ profiling of RNA-RNA spatial interactions. *Nature* **582**, 432–437 (2020). 32499643 10.1038/s41586-020-2249-1

[18] Schmid, M. & Jensen, T. H. Controlling nuclear RNA levels. *Nat. Rev. Genet.* **19**, 518–529 (2018). 29748575 10.1038/s41576-018-0013-2

[19] Lubas, M. et al. Interaction profiling identifies the human nuclear exosome targeting complex. *Mol. Cell* **43**, 624–637 (2011). 21855801 10.1016/j.molcel.2011.06.028

[20] Meola, N. et al. Identification of a nuclear exosome decay pathway for processed transcripts. *Mol. Cell* **64**, 520–533 (2016). 27871484 10.1016/j.molcel.2016.09.025

[21] Ogami, K. et al. An Mtr4/ZFC3H1 complex facilitates turnover of unstable nuclear RNAs to prevent their cytoplasmic transport and global translational repression. *Genes. Dev.* **31**, 1257–1271 (2017). 10.1101/gad.302604.117 PMC5558927 28733371

[22] Contreras, X. et al. PAPgamma associates with the PAXT nuclear exosome to control the abundance of PROMPT ncRNAs. *Nat. Commun.* **14**, 6745 (2023). 37875486 10.1038/s41467-023-42620-9 PMC10598014

[23] Meola, N. & Jensen, T. H. Targeting the nuclear RNA exosome: Poly(A) binding proteins enter the stage. *RNA Biol.* **14**, 820–826 (2017). 28421898 10.1080/15476286.2017.1312227 PMC5546719

[24] Silla, T., Karadoulama, E., Makosa, D., Lubas, M. & Jensen, T. H. The RNA exosome adaptor ZFC3H1 functionally competes with nuclear export activity to retain target transcripts. *Cell Rep.* **23**, 2199–2210 (2018). 29768216 10.1016/j.celrep.2018.04.061 PMC5972229

[25] Yan, J. et al. Histone H3 lysine 4 monomethylation modulates long-range chromatin interactions at enhancers. *Cell Res.* **28**, 204–220 (2018). 29313530 10.1038/cr.2018.1 PMC5799818

[26] Schaaf, C. A. et al. Genome-wide control of RNA polymerase II activity by cohesin. *PLoS Genet.* **9**, e1003382 (2013). 23555293 10.1371/journal.pgen.1003382 PMC3605059

[27] Cubenas-Potts, C. et al. Different enhancer classes in Drosophila bind distinct architectural proteins and mediate unique chromatin interactions and 3D architecture. *Nucleic Acids Res.* **45**, 1714–1730 (2017). 27899590 10.1093/nar/gkw1114 PMC5389536

[28] Liang, J. et al. Chromatin immunoprecipitation indirect peaks highlight long-range interactions of insulator proteins and Pol II pausing. *Mol. Cell* **53**, 672–681 (2014). 24486021 10.1016/j.molcel.2013.12.029 PMC4198380

[29] Rao, S. S. et al. A 3D map of the human genome at kilobase resolution reveals principles of chromatin looping. *Cell* **159**, 1665–1680 (2014). 25497547 10.1016/j.cell.2014.11.021 PMC5635824

[30] Busslinger, G. A. et al. Cohesin is positioned in mammalian genomes by transcription, CTCF and Wapl. *Nature* **544**, 503–507 (2017). 28424523 10.1038/nature22063 PMC6080695

[31] Lai, F. et al. Activating RNAs associate with Mediator to enhance chromatin architecture and transcription. *Nature* **494**, 497–501 (2013). 23417068 10.1038/nature11884 PMC4109059

[32] Rahman, S. et al. Single-cell profiling reveals that eRNA accumulation at enhancer-promoter loops is not required to sustain transcription. *Nucleic Acids Res.* **45**, 3017–3030 (2017). 27932455 10.1093/nar/gkw1220 PMC5389544

[33] Contreras, X. et al. Nuclear RNA surveillance complexes silence HIV-1 transcription. *PLoS Pathog.* **14**, e1006950 (2018). 29554134 10.1371/journal.ppat.1006950 PMC5875879

[34] Salifou, K. et al. Chromatin-associated MRN complex protects highly transcribing genes from genomic instability. *Sci. Adv.* **7**, eabb2947 (2021). 34020942 10.1126/sciadv.abb2947 PMC8139584

[35] Li, H. & Durbin, R. Fast and accurate short read alignment with Burrows-Wheeler transform. *Bioinformatics* **25**, 1754–1760 (2009). 19451168 10.1093/bioinformatics/btp324 PMC2705234

[36] Stadelmayer, B. et al. Integrator complex regulates NELF-mediated RNA polymerase II pause/release and processivity at coding genes. *Nat. Commun.* **5**, 5531 (2014). 25410209 10.1038/ncomms6531 PMC4263189

[37] Wickham, H. *ggplot2: Elegant Graphics for Data Analysis*. (Springer-Verlag, New York, 2016).

[38] Stempor, P. & Ahringer, J. SeqPlots - Interactive software for exploratory data analyses, pattern discovery and visualization in genomics. *Wellcome Open Res*. **1**, 14 (2016). 10.12688/wellcomeopenres.10004.1 PMC5133382 27918597

[39] Kinkley, S. et al. reChIP-seq reveals widespread bivalency of H3K4me3 and H3K27me3 in CD4(+) memory T cells. *Nat. Commun.* **7**, 12514 (2016). 27530917 10.1038/ncomms12514 PMC4992058

[40] Wutz, G. et al. Topologically associating domains and chromatin loops depend on cohesin and are regulated by CTCF, WAPL, and PDS5 proteins. *EMBO J.* **36**, 3573–3599 (2017). 29217591 10.15252/embj.201798004 PMC5730888

[41] Heurteau, A. et al. Insulator-based loops mediate the spreading of H3K27me3 over distant micro-domains, repressing euchromatin genes. *Genome. Biol.* **21**, 193 (2020). 10.1186/s13059-020-02106-z PMC7397589 32746892

[42] Durand, N. C. et al. Juicebox provides a visualization system for Hi-C contact maps with unlimited zoom. *Cell Syst.* **3**, 99–101 (2016). 27467250 10.1016/j.cels.2015.07.012 PMC5596920

[43] Andersson, R. et al. An atlas of active enhancers across human cell types and tissues. *Nature* **507**, 455–461 (2014). 24670763 10.1038/nature12787 PMC5215096

[44] Arner, E. et al. Transcribed enhancers lead waves of coordinated transcription in transitioning mammalian cells. *Science* **347**, 1010–1014 (2015). 25678556 10.1126/science.1259418 PMC4681433

