# Supplementary material for: Nuclear exosome targeting complexes modulate cohesin binding and enhancer-promoter interactions in 3D
Source: Nat Commun. 2026 Aug 6;17:9481. doi: 10.1038/s41467-026-76396-5 (PMC13542147; doi:10.1038/s41467-026-76396-5)
Supplement: Supplementary file 1 [file 41467_2026_76396_MOESM1_ESM.pdf]

# Supplementary Information

**Nuclear exosome targeting complexes modulate cohesin binding and enhancer-promoter interactions in 3D**

Charbel Akkawi[1,#], Alexandre Heurteau[2,3,#], Xavier Contreras[1,#], Marion Helsmoortel[1], Stéphane Schaak[2], Julie Bossuyt[1], Olivier Fosseprez[2], David Dépierre[2], Olivier Cuvier[2*] and Rosemary Kiernan[1*]

[1]Institut de Génétique Humaine (IGH), Univ Montpellier, CNRS, Montpellier, 34396 France

[2] Center of Integrative Biology (CBI), CNRS; Université de Toulouse, France.

[3] Gen2i, Toulouse, France

[#] These authors contributed equally to this work

*Corresponding authors: Rosemary.Kiernan@igh.cnrs.fr; Olivier.Cuvier@utoulouse.fr

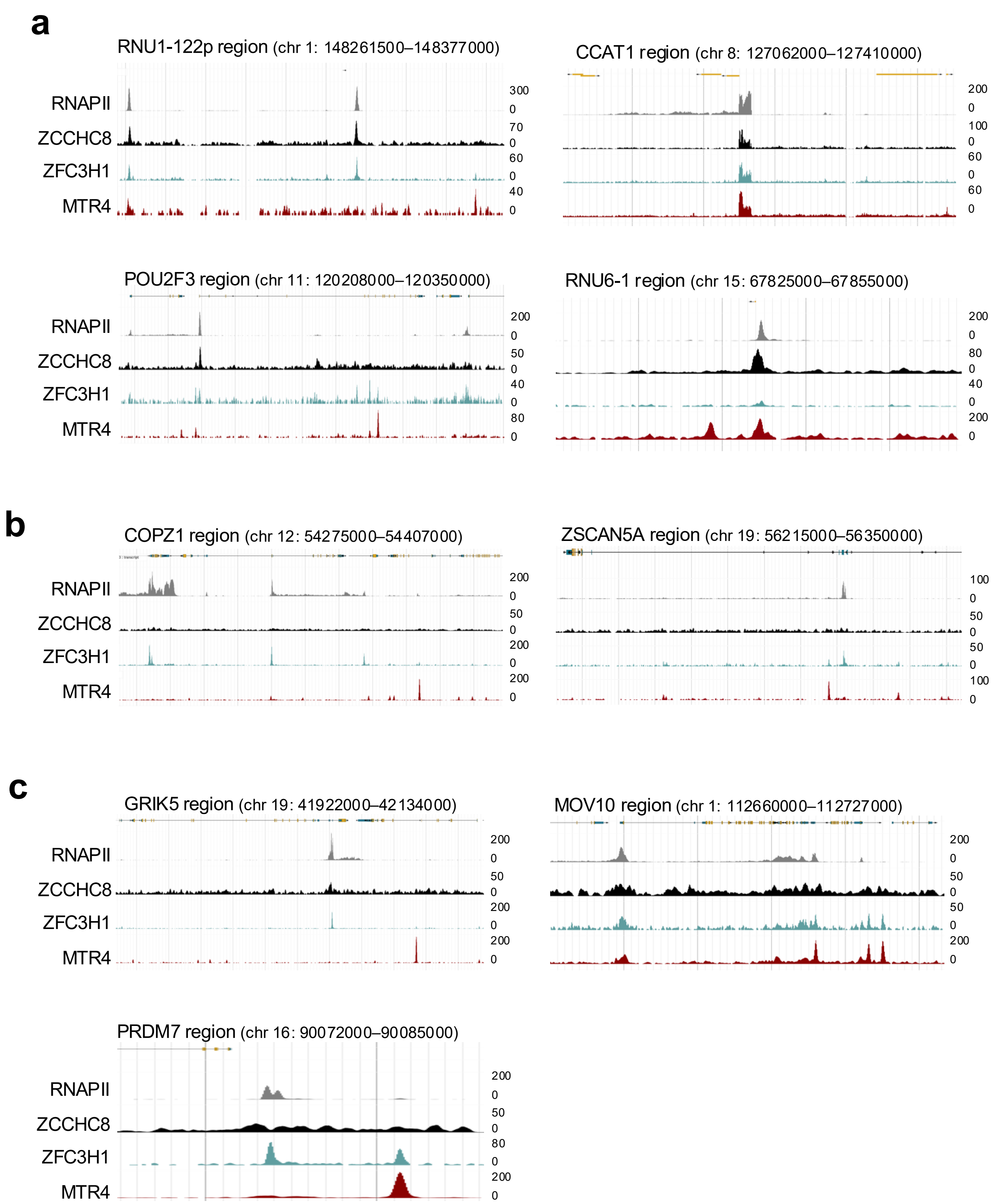
a
RNU1-122p region (chr 1: 148261500–148377000)
RNAPII
ZCCHC8
ZFC3H1
MTR4
300
0
70
0
60
0
40
0
CCAT1 region (chr 8: 127062000–127410000)
200
0
100
0
60
0
60
0
POU2F3 region (chr 11: 120208000–120350000)
200
0
50
0
40
0
80
0
RNU6-1 region (chr 15: 67825000–67855000)
200
0
80
0
40
0
200
0
b
COPZ1 region (chr 12: 54275000–54407000)
200
0
50
0
200
0
200
0
ZSCAN5A region (chr 19: 56215000–56350000)
100
0
50
0
50
0
100
0
c
GRIK5 region (chr 19: 41922000–42134000)
200
0
50
0
200
0
200
0
MOV10 region (chr 1: 112660000–112727000)
200
0
50
0
50
0
200
0
PRDM7 region (chr 16: 90072000–90085000)
200
0
50
0
80
0
200
0

**Supplementary Fig. 1. Genome-wide localization of PAXT, NEXT and MTR4.** Browser shots of RNAPII, ZCCHC8, ZFC3H1 and MTR4 ChIP-seq signal over representative genes in HeLa cells. A schematic representation of the gene is shown above. Shown are representative regions highly associated with ZCCHC8 (**a**), ZFC3H1 (**b**) or MTR4 (**c**).

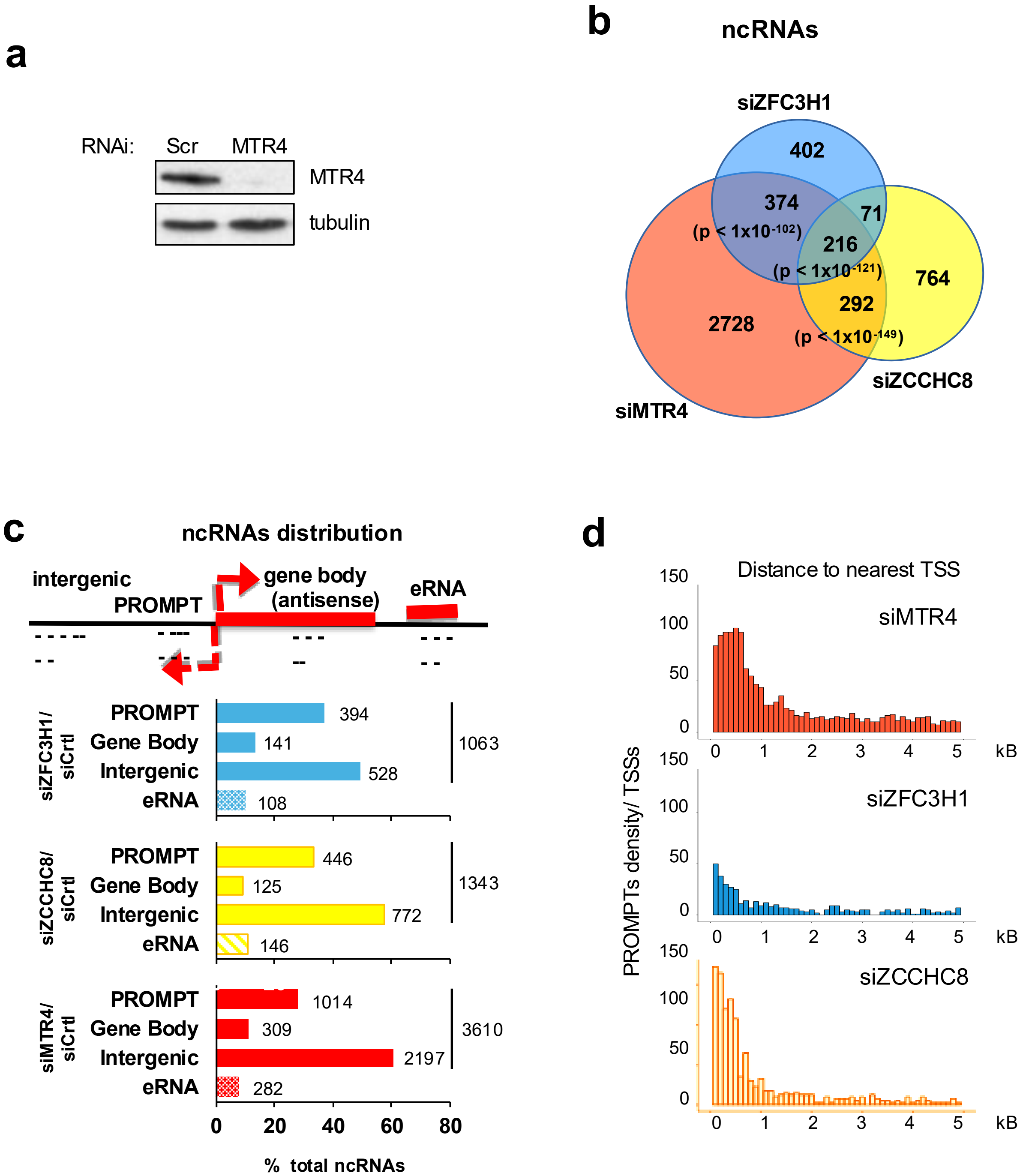
a
RNAi: Scr MTR4
MTR4
tubulin
b
ncRNAs
siZFC3H1
402
374
(p < 1x10-102)
71
216
(p < 1x10-121)
764
292
(p < 1x10-149)
2728
siZCCHC8
siMTR4
c
ncRNAs distribution
intergenic
PROMPT
gene body
(antisense)
eRNA
siZFC3H1/ siCrtl
PROMPT 394
Gene Body 141
Intergenic 528
eRNA 108
1063
siZCCHC8/ siCrtl
PROMPT 446
Gene Body 125
Intergenic 772
eRNA 146
1343
siMTR4/ siCrtl
PROMPT 1014
Gene Body 309
Intergenic 2197
eRNA 282
3610
0 20 40 60 80
% total ncRNAs
d
Distance to nearest TSS
PROMPTs density/ TSSs
siMTR4
siZFC3H1
siZCCHC8
150 100 50 0
0 1 2 3 4 5 kB

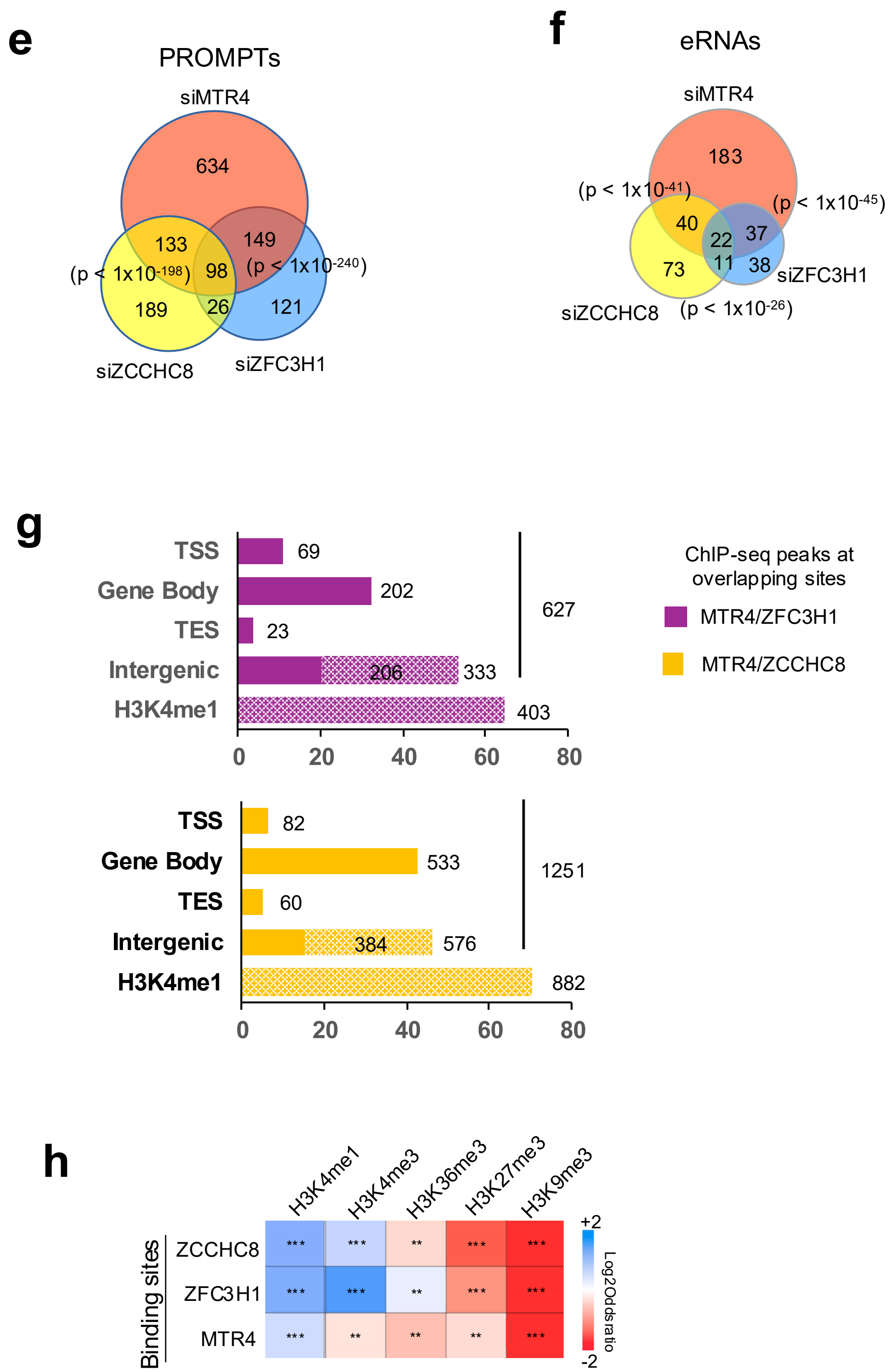
e
PROMPTs
siMTR4
634
133
149
98
(p < 1x10-198)
(p < 1x10-240)
189
26
121
siZCCHC8
siZFC3H1
f
eRNAs
siMTR4
183
(p < 1x10-41)
(p < 1x10-45)
40
22
37
11
38
73
siZFC3H1
siZCCHC8
(p < 1x10-26)
g
TSS
Gene Body
TES
Intergenic
H3K4me1
69
202
23
206
333
403
627
0
20
40
60
80
ChIP-seq peaks at overlapping sites
MTR4/ZFC3H1
MTR4/ZCCHC8
TSS
Gene Body
TES
Intergenic
H3K4me1
82
533
60
384
576
882
1251
0
20
40
60
80
h
H3K4me1
H3K4me3
H3K36me3
H3K27me3
H3K9me3
Binding sites
ZCCHC8
ZFC3H1
MTR4
+2
-2
Log2Odds ratio

**Supplementary Fig. 2. Genome-wide detection of non-coding RNAs and Promoter upstream transcripts (PROMPTs) in cells depleted of MTR4, ZFC3H1 or ZCCHC8.**

**a** Whole cell extracts from HeLa cells treated with siRNAs targeting MTR4 or a non-targeting control, as indicated, were analysed by Western blot using the indicated antibodies. The experiment was performed in triplicate and a representative blot is shown. **b** Venn diagram showing the overlap of ncRNAs that are differentially expressed in cells depleted of MTR4, ZFC3H1, or ZCCHC8 compared with control-depleted cells. P-values were calculated using Fisher's exact test. **c** (top) Schematic representation of categories of ncRNAs detected by differential expression of RNA-seq. (bottom) Histograms depicting the genomic distribution of ncRNAs that are differentially upregulated following depletion of ZFC3H1, ZCCHC8 or MTR4, as indicated, corresponding to gene bodies (antisense transcripts), PROMPTs (antisense transcripts localized within 0 to 2 kb upstream of transcription start sites (TSSs) and intergenic RNAs (ncRNAs present in regions away from -2kb to + 2kb from 5' and 3' end of genes, respectively). Enhancer-associated RNAs (eRNA), which do not correspond to a specific genomic localization, are shown as a hatched bars. **d** Graph showing the distribution of distances between ncRNAs upregulated upon depletion of MTR4, ZFC3H1 or ZCCHC8 and the nearest TSS. **e** Venn diagram showing the overlap of PROMPTs that are differentially expressed (increased) in cells depleted of MTR4, ZFC3H1 or ZCCHC8 compared with control cells. P-values were calculated using Fisher's exact test. **f** Venn diagram showing the overlap of eRNAs that are differentially expressed (increased) in cells depleted of MTR4, ZFC3H1 or ZCCHC8 compared with control cells. P-values were calculated using Fisher's exact test. **g** Histograms showing the genomic distribution of ChIP-seq peaks for ZFC3H1, ZCCHC8, and MTR4 at sites of overlap, as indicated. Peaks are classified according to their localization relative to transcription start sites (TSS), transcription end sites (TES), gene bodies, or intergenic regions (defined as >2 kb from annotated gene units). Peaks overlapping with H3K4me1 (all genomic locations) are indicated by hatched bars. Intergenic peaks overlapping with H3K4me1 are shown as a proportion of all intergenic peaks (hatched). **h** Intersection matrix depicting the relative enrichment of MTR4, ZFC3H1, and ZCCHC8 binding sites with histone post-translational modifications associated with enhancers (H3K4me1), promoters/TSSs (H3K4me3), gene bodies (H3K36me3), or heterochromatin (H3K27me3 and H3K9me3). P-values were calculated by Fisher's exact test (***, $p < 1 \times 10^{-4}$; **, $p < 1 \times 10^{-3}$); log-ratios are indicated by color scale.

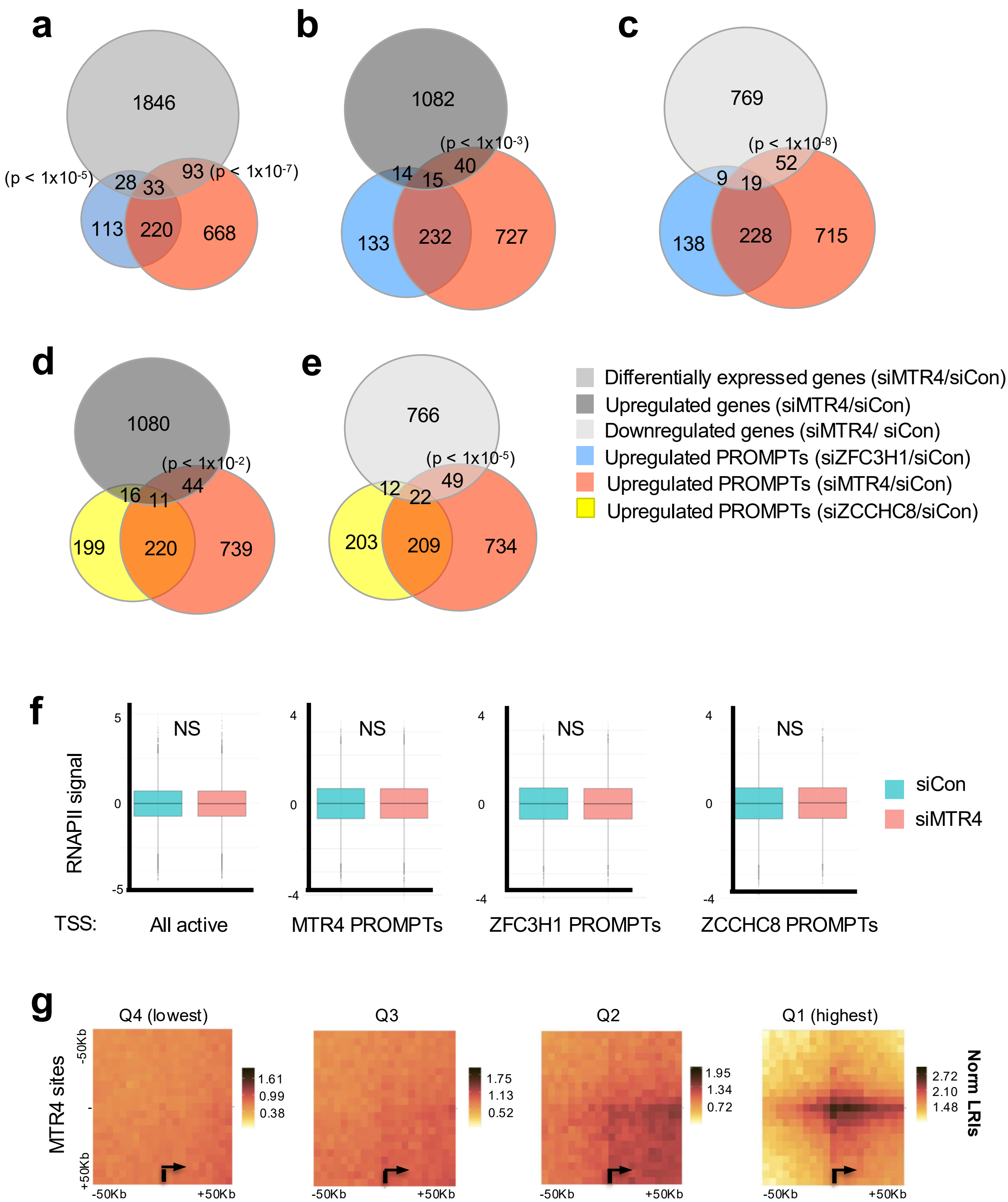

a
1846
(p < 1x10-5)
28
33
93 (p < 1x10-7)
113
220
668
b
1082
(p < 1x10-3)
14
15
40
133
232
727
c
769
(p < 1x10-8)
52
9
19
138
228
715
d
1080
(p < 1x10-2)
44
16
11
199
220
739
e
766
(p < 1x10-5)
49
12
22
203
209
734
Differentially expressed genes (siMTR4/siCon)
Upregulated genes (siMTR4/siCon)
Downregulated genes (siMTR4/ siCon)
Upregulated PROMPTs (siZFC3H1/siCon)
Upregulated PROMPTs (siMTR4/siCon)
Upregulated PROMPTs (siZCCHC8/siCon)
f
RNAPII signal
NS
siCon
siMTR4
TSS:
All active
MTR4 PROMPTs
ZFC3H1 PROMPTs
ZCCHC8 PROMPTs
g
MTR4 sites
Q4 (lowest)
Q3
Q2
Q1 (highest)
-50Kb
+50Kb
1.61
0.99
0.38
1.75
1.13
0.52
1.95
1.34
0.72
2.72
2.10
1.48
Norm LRIs

**Supplementary Fig. 3. MTR4 influences PROMPTs through long-range interactions.**

**a-e** Venn diagrams show the overlap between differentially expressed PROMPTs detected after depletion of MTR4, ZFC3H1, or ZCCHC8 and genes whose expression was either differentially expressed, upregulated, or downregulated upon MTR4 loss (FDR <0.05). *P* values were calculated using Fisher's exact test. **f** Box plots showing RNAPII ChIP-seq signal at TSSs of all active genes, or at TSSs that accumulate PROMPTs after depletion of MTR4, ZFC3H1 or ZCCHC8, as indicated. Statistical significance was assessed using Wilcoxon's test (NS, not significant). **g** 2D APA plots representing normalized Hi-C counts at enhancers bound by MTR4 and active TSSs within 1 Mbp of an active enhancer. Aggregation analysis was stratified by quartiles containing the highest number of contacts (Q1) to the lowest number of contacts (Q4).

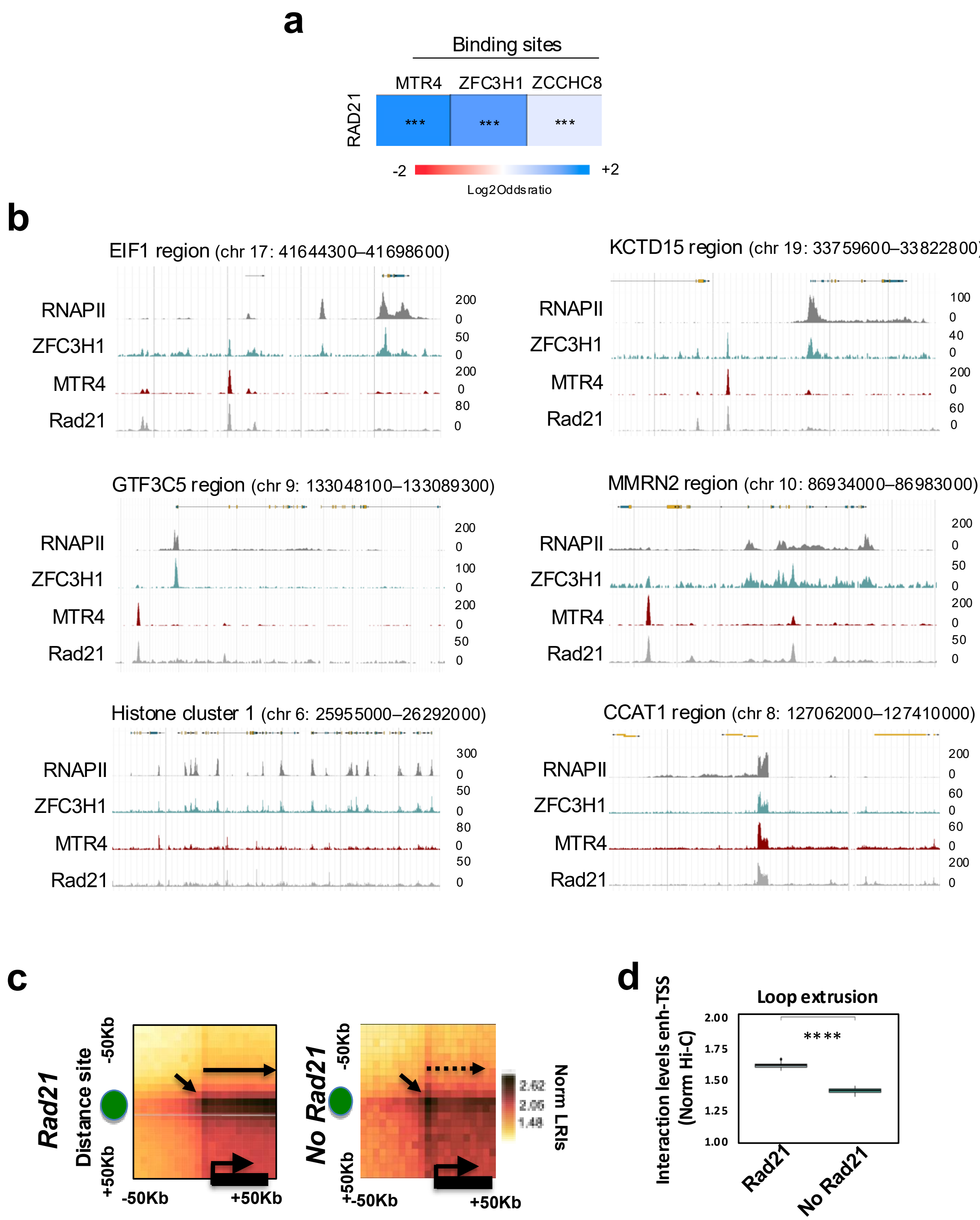
a
Binding sites
MTR4
ZFC3H1
ZCCHC8
RAD21
***
***
***
-2
+2
Log2Oddsratio
b
EIF1 region (chr 17: 41644300–41698600)
KCTD15 region (chr 19: 33759600–33822800)
GTF3C5 region (chr 9: 133048100–133089300)
MMRN2 region (chr 10: 86934000–86983000)
Histone cluster 1 (chr 6: 25955000–26292000)
CCAT1 region (chr 8: 127062000–127410000)
RNAPII
ZFC3H1
MTR4
Rad21
c
Rad21
No Rad21
Distance site
-50Kb
+50Kb
Norm LRIs
2.62
2.05
1.48
d
Loop extrusion
Interaction levels enh-TSS
(Norm Hi-C)
****
2.00
1.75
1.50
1.25
1.00
Rad21
No Rad21

**Supplementary Fig. 4. MTR4 colocalizes with cohesin.**

**a** Intersection matrix showing the relative enrichments of the binding sites of MTR4, ZFC3H1 and ZCCHC8 with those of Rad21. P-values were calculated by Fisher's exact test (***, $p < 1 \times 10^{-4}$); log-ratios are indicated by color scale. **b** Browser shots of RNAPII, ZFC3H1, MTR4 and Rad21 ChIP-seq signal over representative regions in HeLa cells. A schematic representation of the region is shown above. **c** 2D APA plots representing normalized Hi-C counts at enhancers bound by Rad21 or not (left and right plot, respectively). Aggregation analysis was performed between enhancers and all active TSSs within 1 Mbp. **d** Box plot showing the distribution of long-range interactions along the bodies of genes contacted by distant enhancers depending on the presence or absence of Rad21. The statistical variations were tested by Wilcoxon test (****, $p < 1 \times 10^{-6}$).

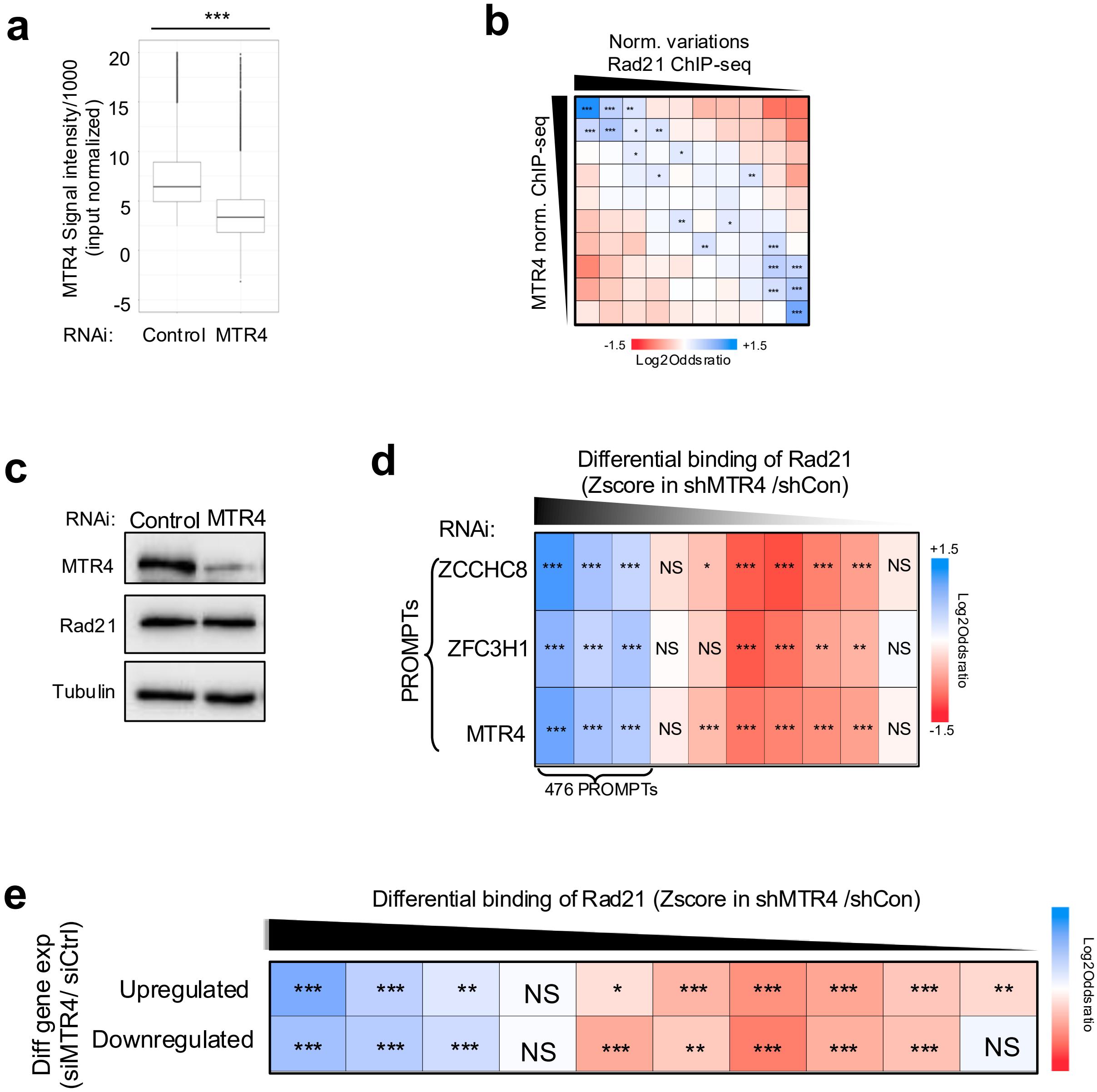

a
***
MTR4 Signal intensity/1000
(input normalized)
20
15
10
5
0
-5
RNAi:
Control
MTR4
b
Norm. variations
Rad21 ChIP-seq
MTR4 norm. ChIP-seq
-1.5
+1.5
Log2Oddsratio
c
RNAi:
Control
MTR4
MTR4
Rad21
Tubulin
d
Differential binding of Rad21
(Zscore in shMTR4 /shCon)
RNAi:
PROMPTs
ZCCHC8
*** *** *** NS * *** *** *** *** NS
ZFC3H1
*** *** *** NS NS *** *** ** ** NS
MTR4
*** *** *** NS *** *** *** *** *** NS
476 PROMPTs
+1.5
-1.5
Log2Oddsratio
e
Differential binding of Rad21 (Zscore in shMTR4 /shCon)
Diff gene exp
(siMTR4/ siCtrl)
Upregulated
*** *** ** NS * *** *** *** *** **
Downregulated
*** *** *** NS *** ** *** *** *** NS
Log2Oddsratio

**Supplementary Fig. 5 MTR4 regulates cohesin association at sites of long-range chromatin interactions.**

**a** Box plots of MTR4 ChIP-seq reads at regions bound by MTR4 in cells treated with shMTR4 or shControl, as indicated (***, $p < 1 \times 10^{-4}$, Wilcoxon test). **b** Intersection matrix showing Rad21 binding, stratified by the net variation in MTR4-depleted compared to control cells (horizontal axis), and the same sites stratified by MTR4 binding levels (vertical axis). The enrichment with distant H3K4me1 sites (not overlapping a TSS) was calculated using a Fisher's exact test (***, $p < 1 \times 10^{-4}$; **, $p < 1 \times 10^{-3}$; *, $p < 1 \times 10^{-2}$). **c** Whole cell extracts from HeLa cells treated with shRNA targeting MTR4 or a nontargeting control, as indicated, were analysed by Western blot using antibodies against Rad21 or tubulin. The experiment was performed in duplicate and a representative blot is shown. **d** Intersection matrix showing Rad21 binding, stratified by net variation upon MTR4 depletion compared to control, and increased or decreased gene expression, as indicated. The enrichment with PROMPTs was calculated using Fisher's exact test (***, $p < 1 \times 10^{-4}$; **, $p < 1 \times 10^{-3}$; *, $p < 1 \times 10^{-2}$; NS = Not significant). **e** Intersection matrix showing Rad21 binding, stratified by net variation upon MTR4 depletion compared to control, and increased or decreased gene expression, as indicated. The enrichment with PROMPTs was calculated using a Fisher's exact test (***, $p < 1 \times 10^{-4}$; **, $p < 1 \times 10^{-3}$; *, $p < 1 \times 10^{-2}$; NS = Not significant).

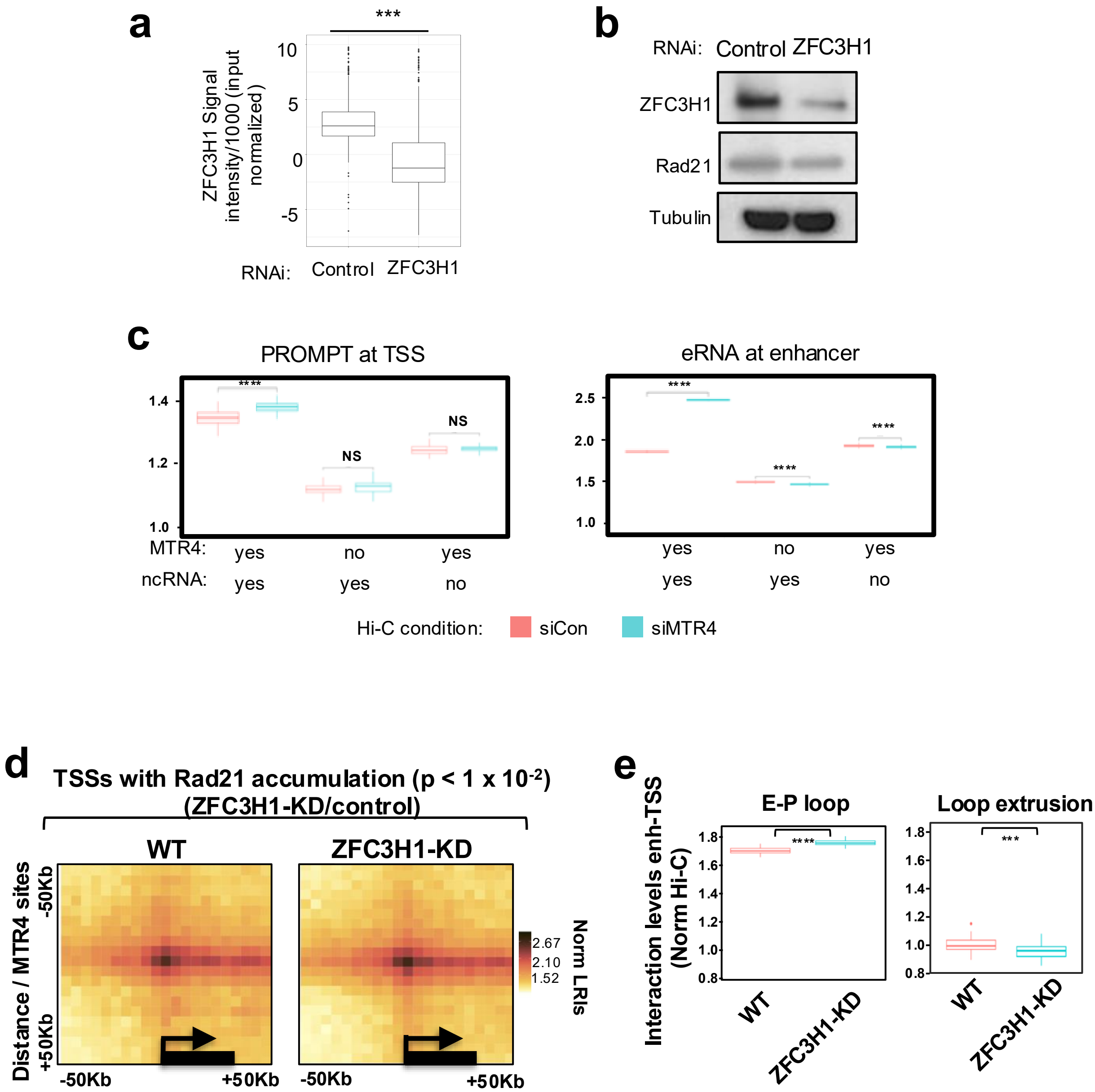

a
***
ZFC3H1 Signal intensity/1000 (input normalized)
10
5
0
-5
RNAi: Control ZFC3H1
b
RNAi: Control ZFC3H1
ZFC3H1
Rad21
Tubulin
c
PROMPT at TSS
eRNA at enhancer
****
NS
NS
1.4
1.2
1.0
2.5
2.0
1.5
1.0
MTR4: yes no yes
ncRNA: yes yes no
Hi-C condition: siCon siMTR4
d
TSSs with Rad21 accumulation (p < 1 x 10-2)
(ZFC3H1-KD/control)
WT
ZFC3H1-KD
Distance / MTR4 sites
-50Kb
+50Kb
2.67
2.10
1.52
Norm LRIs
e
Interaction levels enh-TSS (Norm Hi-C)
E-P loop
Loop extrusion
****
***
1.8
1.6
1.4
1.2
1.0
0.8
WT
ZFC3H1-KD

**Supplementary Fig. 6 Nuclear exosome targeting complexes regulate cohesion association and long-range chromatin interactions.**

**a** Box plots of ZFC3H1 ChIP-seq reads at regions bound by ZFC3H1 in cells treated with shZFC3H1 or shControl, as indicated (***, $p < 1 \times 10^{-4}$, two-sided Wilcoxon test). **b** Whole cell extracts from HeLa cells treated with shRNA targeting ZFC3H1 or a nontargeting control, as indicated, were analysed by Western blot using antibodies against Rad21, ZFC3H1 or tubulin. The experiment was performed in duplicate and a representative blot is shown **c** Box plots showing the distribution of LRIs at E-P contacts, in control (red) or MTR4-depleted (green) conditions at sites that are bound or not by MTR4 in control cells, and at sites that accumulating PROMPTs or eRNAs following depletion of MTR4, as indicated. The statistical variations were tested by Wilcoxon test (****, $p < 1 \times 10^{-6}$; NS, not significant. **d** 2D APA plots representing normalized Hi-C counts at distant MTR4 sites in control or ZFC3H1-depleted conditions (left and right plot, respectively). Aggregation analysis was performed between enhancers and all active TSSs within 1 Mbp. **e** Box plot showing the distribution of LRIs at interaction sites (E-P loop, left box plot) or along the bodies of genes contacted by distant enhancers (loop extrusion, right box plot) in control or ZFC3H1-depleted conditions, as indicated. The statistical variations were tested by Wilcoxon test (****, $p < 1 \times 10^{-6}$; ***, $p < 1 \times 10^{-4}$).

**Supplementary Table 1.** Antibodies used in this study.

| Antibody | Reference | Supplier |
|---|---|---|
| **MTR4 (ChIP-seq)** | Ab70552 | Abcam |
| **MTR4 (WB)** | A300-614A | Bethyl laboratories |
| **ZFC3H1 (ChIP-seq)** | A301-457A | Bethyl laboratories |
| **ZFC3H1 (WB)** | A301-456A | Bethyl laboratories |
| **ZCCHC8 (ChIP-seq)** | A301-805A | Bethyl laboratories |
| **ZCCHC8 (WB)** | A301-806A | Bethyl laboratories |
| **RNAPII (F-12) (ChIP-seq)** | 55492 | SCBT |
| **Rad21 (ChIP-seq and WB)** | Ab992 | Abcam |
| **TUBULIN** | T9026 | Sigma-Aldrich |

**Supplementary Table 2. Sequences of siRNAs used in this study.**

| siRNA | Sequence (5' to 3') |
|---|---|
| Control | ucugcaagguuaggcgucu (dTdT) |
| MTR4 | acacugagcuggaaaauaa(dTdT) |
| ZFC3H1 | cuauagcaguccaucuuaa(dTdT) |
