# Supplementary material for: Nuclear exosome targeting complexes modulate cohesin binding and enhancer-promoter interactions in 3D
Source: Nat Commun. 2026 Aug 6;17:9481. doi: 10.1038/s41467-026-76396-5 (PMC13542147; doi:10.1038/s41467-026-76396-5)
Supplement: Supplementary file 3 [file 41467_2026_76396_MOESM3_ESM.pdf]

nature portfolio

Peer Review File

Nuclear exosome targeting complexes modulate cohesin binding and enhancer-promoter interactions in 3D

Corresponding Author: Dr Rosemary Kiernan

This file contains all reviewer reports in order by version, followed by all author rebuttals in order by version.

Version 0:

Reviewer comments:

Reviewer #1

(Remarks to the Author)
Akkawi et al. investigated the chromatin binding of the NEXT subunit ZCCHC8, the shared helicase MTR4, and the PAXT subunit ZFC3H1. They found that ZCCHC8 and ZFC3H1 predominantly bind to transcription start sites (TSS) and enhancers. The shRNA knockdown of MTR4 resulted in increased signals for eRNAs and PROMPTs, despite MTR4 being largely absent at TSSs. By integrating Hi-C data, the authors proposed that MTR4 contacts TSSs through 3D chromatin interactions between enhancers and promoters. Furthermore, they showed that MTR4 modulates Cohesin chromatin binding. Collectively, these findings implicate that RNA surveillance factors play a role in organizing Cohesin-mediated 3D genome architecture.

However, there are several issues with this manuscript that prevent me from supporting its publication. First, while the connections between RNA surveillance factors and chromatin organization have been established, the key point of this manuscript lacks novelty. Previous studies have shown that defects in nuclear RNA homeostasis lead to RNA aggregates and various transcriptional, translational, and chromatin organizational defects. This results in difficulties when interpreting the observations made after prolonged knockdown of nuclear RNA exosome-associated factors, making it unclear whether the observed effects are direct or indirect. Furthermore, the molecular mechanism by which MTR4 regulates Cohesin chromatin binding remains unclear.

To address potential secondary effects of shRNA knockdown, the authors should perform RNA-seq analyses, Pol II ChIP-seq, and ribosome profiling. Previous research has indicated that dysregulation of RNA surveillance can adversely affect Pol I and Pol II transcription as well as translation (PMID: 38631356). Additionally, to capture immediate perturbations, acute protein degradation analyses would be beneficial.

Figure 1: Given that previous studies have shown that ZCCHC8 regulates retrotransposons (PMID: 31747613; PMID: 35349793), the authors should compare their chromatin binding data with published datasets to determine if their binding includes retrotransposon elements. It would also be insightful to explore whether the effects on 3D genome organization are linked to the dysregulation of retrotransposons.

The overlap among the chromatin binding profiles of MTR4, ZCCHC8, and ZFC3H1 is minimal. Does this indicate functional diversity or moonlighting functions for these factors? If so, it is critical to conduct analyses using enzymatic mutants to clarify the specific roles of the nuclear exosome.

As the authors claim effects on enhancer-promoter interactions, they should provide evidence of specific loops formed between enhancers and promoters. Since Hi-C resolution is low, high-resolution 4C analysis would be advantageous. Additionally, snapshots of enhancer and promoter binding signals, as well as changes in specific PROMPTs, should be presented.

What is the specificity of MTR4's effects on enhancer-promoter interactions? Can the authors analyze chromatin structure data post-knockdown by subgrouping based on MTR4 binding presence/absence and its effects on PROMPTs? Additionally, given that the authors possess RNA-seq data, do the genes affected by PROMPTs exhibit changes in expression? Are these differentially expressed genes enriched in specific functional pathways?

In Figure 4, the authors show that the knockdown of ZFC3H1 leads to an increase in RAD21. Does this knockdown also induce defects in 3D chromatin organization? Moreover, if ZFC3H1 influences PROMPTs at TSSs, how does it concurrently affect RAD21? Lastly, does MTR4 have any effect on enhancer RNAs?

Reviewer #2

(Remarks to the Author)
This study comprehensively investigates the role of RNA Exosome targeting complexes in regulating three-dimensional (3D) chromatin structure. The findings reveal notable discrepancies in genome occupancy among NEXT, PAXT, and the RNA helicase MTR4, despite the helicase's established role in cooperating with both NEXT and PAXT. Furthermore, MTR4 knockdown via RNA interference (RNAi) resulted in increased cohesin levels, particularly an elevated Rad21 signal at chromatin loops. Based on these observations, the authors propose that the RNA helicase MTR4 regulates enhancer-promoter interactions by modulating the levels of non-coding RNAs (ncRNAs).
However, further clarification and additional experimental evidence are necessary to substantiate these claims. As MTR4 operates in concert with NEXT, PAXT, and the RNA Exosome complex, isolating the specific role of MTR4 from its interactions with these complexes is inherently challenging. To address this issue, it is essential to examine the effects of MTR4 knockdown in a context where NEXT or PAXT depletion does not interfere. For instance, identifying ncRNAs exclusively affected by MTR4 knockdown, but not by NEXT or PAXT depletion, would help delineate the MTR4-specific effects.
In this context, relying solely on RNA-seq after prolonged (72-hour) depletion of each component may not provide adequate resolution to distinguish their specific targets. Instead, additional experimental approaches, such as nascent RNA analysis or CLIP-seq, would be more suitable for capturing real-time transcriptional activity and identifying the direct binding sites of these complexes.
The following points are to be reconsidered:
First, the authors have repeatedly used the terms "nuclear exosome targeting complexes" and "nuclear RNA exosome" interchangeably throughout the manuscript. However, these are distinct protein complexes with fundamentally different roles. It is recommended to use the term "nuclear exosome targeting complexes/helicase" specifically when referring to NEXT, PAXT, and MTR4 to avoid confusion and ensure terminological precision.
There are several typographical errors and figure number misrepresentations throughout the manuscript that need to be addressed for clarity and accuracy:
Line 55: Replace "(TADS)" with the correct term "TADs."
Line 215: Replace "Aggregated plot analysis" with the term "Aggregated peak analysis"
Line 251: Update "Fig. 5c" to "Fig. 4c."
Line 273: Correct "Supplementary Fig. 6A" to "Fig. 5a."
Line 294: Update "Fig.5D" to "Fig.5d"
Add references to following descriptions are recommended
Line62, Line67
In Figure 5b, to avoid potential confusion, it is recommended to use the term "chromatin loop" (or "EP loop") instead of "Central area." This terminology would provide greater clarity and align with commonly accepted nomenclature in the field.
In Figure 5d, although the figure represents the condition of MTR4 depletion, the presence of MTR4 is still depicted. Additionally, the transcription of PROMPTs appears to increase, as indicated by the red arrow. Is there any evidence supporting the claim that MTR4 knockdown (KD) leads to increased transcription of PROMPTs? If the primary effect is ncRNA accumulation rather than enhanced transcription, the figure should depict a red arrow of the same size accompanied by an increased quantity of RNAs. Finally, to accurately reflect the observed accumulation of Rad21, it would be more appropriate to illustrate an increased number of cohesin rings in the MTR4-KD condition.
In the discussion, the proposed model suggests that accumulated ncRNAs may stabilize cohesin loops. However, have you also considered the possibility that the observed increase in Rad21 at these regions could be attributed not to ncRNAs, but rather to changes in chromatin accessibility or region availability following MTR4 depletion? Exploring this alternative explanation could provide additional insights into the underlying mechanisms.
Further Recommendations for Figure Representations:
1. Resolution Issues: Several figures have low resolution, making them appear blurred. Improving the resolution of these figures is essential to ensure clarity in data presentation.
2. Figure 1(b): The representation of p-values should be corrected for consistency and proper formatting. For instance, change "1e-126" to "1 × 10^-126" or "1e-126."
3. Figure 1(c-e): Include an explanation for the p-value representation (***) in the figure legend or directly within the figure. This clarification is necessary for readers to interpret the significance levels accurately.
4. Figure 2(b): In the left-bottom Venn diagram, the sum of MTR4 peaks is reported as 5672, while in other figures, the sum is 5666. Please clarify the discrepancy or provide an explanation for the difference.
5. Figure 5(c): Represent the size of the Hi-C matrix in base pairs (bp) to provide a more comprehensive understanding of the data resolution. Adding this detail will enhance the interpretability of the figure.
I strongly believe addressing these points would enhance this study's comprehensibility, accuracy, and validity.

Reviewer #3

(Remarks to the Author)
In their manuscript "Nuclear exosome targeting complexes modulate cohesin binding and enhancer-promoter interactions in

3D”, Akkawi et al. implicate exosome targeting complexes in the regulation of 3D genome contacts. This is supported by data demonstrating the localization of exosome targeting factors to DNA contact points (in particular enhancers and TSS) and chromatin conformation capture experiments comparing targeting factor knockdown and controls. They further provide evidence that the changes in genome conformation are reflected in altered cohesion levels at the contact sites where exosome targeting factors are present.
This is an interesting, well-conducted study highlighting the responsiveness of the genome to the non-coding transcriptome and the role of nuclear RNA surveillance factors in shaping this landscape.

General comments:
The figures are rich in information, and additional details in the figure legends would sometimes aid in interpreting the data (see below).
In the discussion, the authors state: “It seems unlikely that MTR4 or associated complexes, such as PAXT, are directly responsible for the effects observed on LRIs. Instead, it seems more probable that ncRNA targets of nuclear exosome, particularly eRNAs and PROMPTs, that were concomitantly stabilized at the enhancers and promoters in contact, may be responsible for the effect on LRIs and loop extrusion.”. This s a very reasonable assumption. But - since the authors have both RNA expression and chromosome conformation data available for control and targeting factor knockdown – why not use the available data to explore correlations that may directly support that idea? This was done for PROMPTs to some extent, but not for eRNA.

Minor points:
Figure 2a shows the distribution of ChIP-seq peaks across different features, including TSS, TES, gene bodies and intergenic. Later in the manuscript, the main focus is on TSS and enhancers. I assume enhancers are part of the intergenic features. Would it be possible to include the information in this figure, e.g. as percentage of intergenic peaks that correspond to enhancers (and maybe also percentage of TSS that are associated with a PROMPT)? It might also be good to include absolute numbers for the counted peaks in each category. Alternatively, a metagene analysis as in Fig 1c-e could be repeated split into peaks associated with the different features?
Figure 2b: It would be interesting to know whether the overlap of ChIP peaks between samples is determined by the features that the peaks are found at. Would it be possible to integrate that information in figure 2b, e.g. by including miniatures of bar plots similar to the ones shown in 2a?
I am confused about the labelling of some bubbles in Fig 2b, and Supp. Fig 3 (all panels) as “PROMPTs DETECTED in cells depleted…”. What is the cut-off for detection? Why not show the ones that are differentially expressed upon knock-down (and state the cut-off for that)?
Line 176/177: “In contrast, MTR4 recruitment sites were enriched in the histone H3K4me1 mark that is associated with enhancers” – To me, this sound as if Z3 and Z8 were not enriched at enhancers, but they are (even more strongly than MTR4 according to Supp Fig 2G). Please change the wording accordingly.
Abstract: “While nuclear RNA exosome, … control”   “While the nuclear RNA exosome, … controls”
Line 162/163: “previous analyses showing the contribution of MTR4-containing complexes in regulating ncRNAs including eRNAs and PROMPTs”   please include references
In Supp. Fig 3A, B, and C, the numbers of genes do not fully add up (B + C is not equal to A).
Line 203/204: “LRIs with distant TSSs that accumulate PROMPTs upon nuclear exosome depletion” – To me, the wording suggests that the PROMPTs are retained on the TSS at the chromatin. Please change in a way that it is clear you refer to RNA levels.
Lines 281, 286, 291, 292 and others: “nuclear RNA exosome complexes”   “nuclear RNA exosome targeting complexes”
Lines 406-408: “Preliminary analyses were performed in comparison with…”.   Was this included in the manuscript? If not, remove?
Figure 3b: To make the browser shots easier to interpret, it would be helpful if relevant features (TSS, enhancers) could be marked. Potentially combine with browser shots for RNAseq data (siMTR4, siZ3) for the location of PROMPTs?
Figure 3c: Please specify how “enrichment in PROMPTs” was determined. Is this the log2FC for reads in siMTR4 vs control? Does this graph include LRI scores for all enhancer-TSS contacts genome-wide (in that case, I would expect at least the three lower quartiles to reflect zero interactions), or was this analysis performed on a window as stated for Figure 4a (“all active TSSs within 1Mbp”)? Please specify. Out of interest, could you also include summary stats for LRIs in the different quartiles for MTR4-bound and non-bound enhancers (in the supplements?). This could reveal a general trend of the impact of the presence of MTR4 on LRIs.
Figure 3d: Scale in grayscale, figure uses a coloured scale. Arrows almost point to the centre of the plot, but not quite. Is that intended?
Fig 5a & Suppl. 4C: Please include scale.

Version 1:

Reviewer comments:

Reviewer #1

(Remarks to the Author)
The authors have primarily addressed my comments by expanding the discussion, with limited new experimental data. If the authors agree that the novelty of the manuscript and the potential for indirect effects remain as concerns, I recommend performing acute protein degradation experiments and related RNA-seq analayses to better exclude indirect effects.

On one hand, the authors have not provided high-resolution data on E–P conformational changes; on the other, they have not clarified how ZFC3H1 regulates RAD21. Thus, the current evidence supporting the extension of the conclusion to Cohesin binding and E–P interactions is insufficient for publication in Nature Communications.

Reviewer #2

(Remarks to the Author)
I have carefully reviewed the authors' revised manuscript and the point-by-point response to the reviewers. The authors have provided clear experimental evidence, expanded explanations, and appropriate revisions throughout the manuscript, which collectively strengthen the scientific rigor and clarity of the work. Therefore, I recommend the manuscript for acceptance in its current form.

Reviewer #3

(Remarks to the Author)
All points that I have raised have been satisfactorily addressed in the revision.

Open Access This Peer Review File is licensed under a Creative Commons Attribution 4.0 International License, which permits use, sharing, adaptation, distribution and reproduction in any medium or format, as long as you give appropriate credit to the original author(s) and the source, provide a link to the Creative Commons license, and indicate if changes were made.
In cases where reviewers are anonymous, credit should be given to 'Anonymous Referee' and the source.
The images or other third party material in this Peer Review File are included in the article's Creative Commons license, unless indicated otherwise in a credit line to the material. If material is not included in the article's Creative Commons license and your intended use is not permitted by statutory regulation or exceeds the permitted use, you will need to obtain permission directly from the copyright holder.
To view a copy of this license, visit https://creativecommons.org/licenses/by/4.0/

Reviewer #1

Akkawi et al. investigated the chromatin binding of the NEXT subunit ZCCHC8, the shared helicase MTR4, and the PAXT subunit ZFC3H1. They found that ZCCHC8 and ZFC3H1 predominantly bind to transcription start sites (TSS) and enhancers. The shRNA knockdown of MTR4 resulted in increased signals for eRNAs and PROMPTs, despite MTR4 being largely absent at TSSs. By integrating Hi-C data, the authors proposed that MTR4 contacts TSSs through 3D chromatin interactions between enhancers and promoters. Furthermore, they showed that MTR4 modulates Cohesin chromatin binding. Collectively, these findings implicate that RNA surveillance factors play a role in organizing Cohesin-mediated 3D genome architecture.

However, there are several issues with this manuscript that prevent me from supporting its publication. First, while the connections between RNA surveillance factors and chromatin organization have been established, the key point of this manuscript lacks novelty. Previous studies have shown that defects in nuclear RNA homeostasis lead to RNA aggregates and various transcriptional, translational, and chromatin organizational defects. This results in difficulties when interpreting the observations made after prolonged knockdown of nuclear RNA exosome-associated factors, making it unclear whether the observed effects are direct or indirect. Furthermore, the molecular mechanism by which MTR4 regulates Cohesin chromatin binding remains unclear.
To address potential secondary effects of shRNA knockdown, the authors should perform RNA-seq analyses, Pol II ChIP-seq, and ribosome profiling.

Response:

We appreciate the reviewer’s concerns regarding novelty and potential indirect effects. Our study extends previous work by directly linking nuclear exosome targeting factors to modulation of Cohesin binding and enhancer–promoter interactions. To minimize secondary consequences of prolonged knockdown, all experiments were performed at early time points and cells were harvested before the onset of overt toxicity. Consistent with a primary post-transcriptional effect, RNAPII ChIP-seq show that PROMPT accumulation is not accompanied by increased RNAPII occupancy at the corresponding loci (Supplementary Fig. 3F), arguing against indirect transcriptional upregulation.

We agree that MTR4 depletion has been associated with translation defects (Ogami et al., 2017), but these effects arise at later stages than those examined in our study. While acute degron approaches would indeed be valuable for dissecting immediate functions, such tools are not yet available for MTR4 in our system. Importantly, the chromatin and transcriptomic effects we report were reproduced using two independent knockdown strategies (siRNA and shRNA) and across two components of the PAXT complex (MTR4 and ZFC3H1), supporting the robustness and specificity of our conclusions.

Previous research has indicated that dysregulation of RNA surveillance can adversely affect Pol I and Pol II transcription as well as translation (PMID: 38631356). Additionally, to capture immediate perturbations, acute protein degradation analyses would be beneficial.

Response:

We appreciate the reviewer's reference to a recent study by Han et al., carried out in iPS cells employing acute degradation of EXOSC2 (PMID: 38631356). While highly informative, these experiments target a core exosome subunit that is distributed throughout the nucleoplasm, nucleolus, and cytoplasm. In contrast, MTR4 and ZFC3H1, the factors targeted in our study, are nucleoplasmic. EXOSC2 depletion therefore induces broader perturbations, including a substantial impact on rRNA synthesis, compared to the more specific effects of MTR4 depletion in our system. We agree that acute protein degradation would be ideal, but such systems are currently not available for MTR4.

Figure 1: Given that previous studies have shown that ZCCHC8 regulates retrotransposons (PMID: 31747613; PMID: 35349793), the authors should compare their chromatin binding data with published datasets to determine if their binding includes retrotransposon elements. It would also be insightful to explore whether the effects on 3D genome organization are linked to the dysregulation of retrotransposons.

Response:

We agree this is an important point. We therefore examined whether cohesin redistribution following MTR4 depletion overlaps with transposable elements (Rev figure 1). Cohesin binding was not significantly changed at LINE1 elements, previously reported as HUSH targets. On the other hand, it was significantly changed at sites of several TE families. Cohesin binding changed at Alu elements, which is notable given the recent report by Liang et al. (2023), showing that Alu elements can mediate enhancer–promoter interactions. Changes were also measured at LINE2 elements and MIRs. Although no longer active, sequences derived from both of these TE families influence gene regulation and genome architecture.

Thus, the analysis suggests that ZCCHC8 regulation of HUSH targets is not a contributing factor in the observed changes in genome folding. The changes in cohesin binding at LINE2 and MIR sites further strengthen our conclusion that depletion of MTR4 is involved in the regulation of genome folding at the expected sites.

This analysis has not been included in the manuscript, but we would be happy to add it to the supplementary data should the reviewer find it sufficiently enlightening.

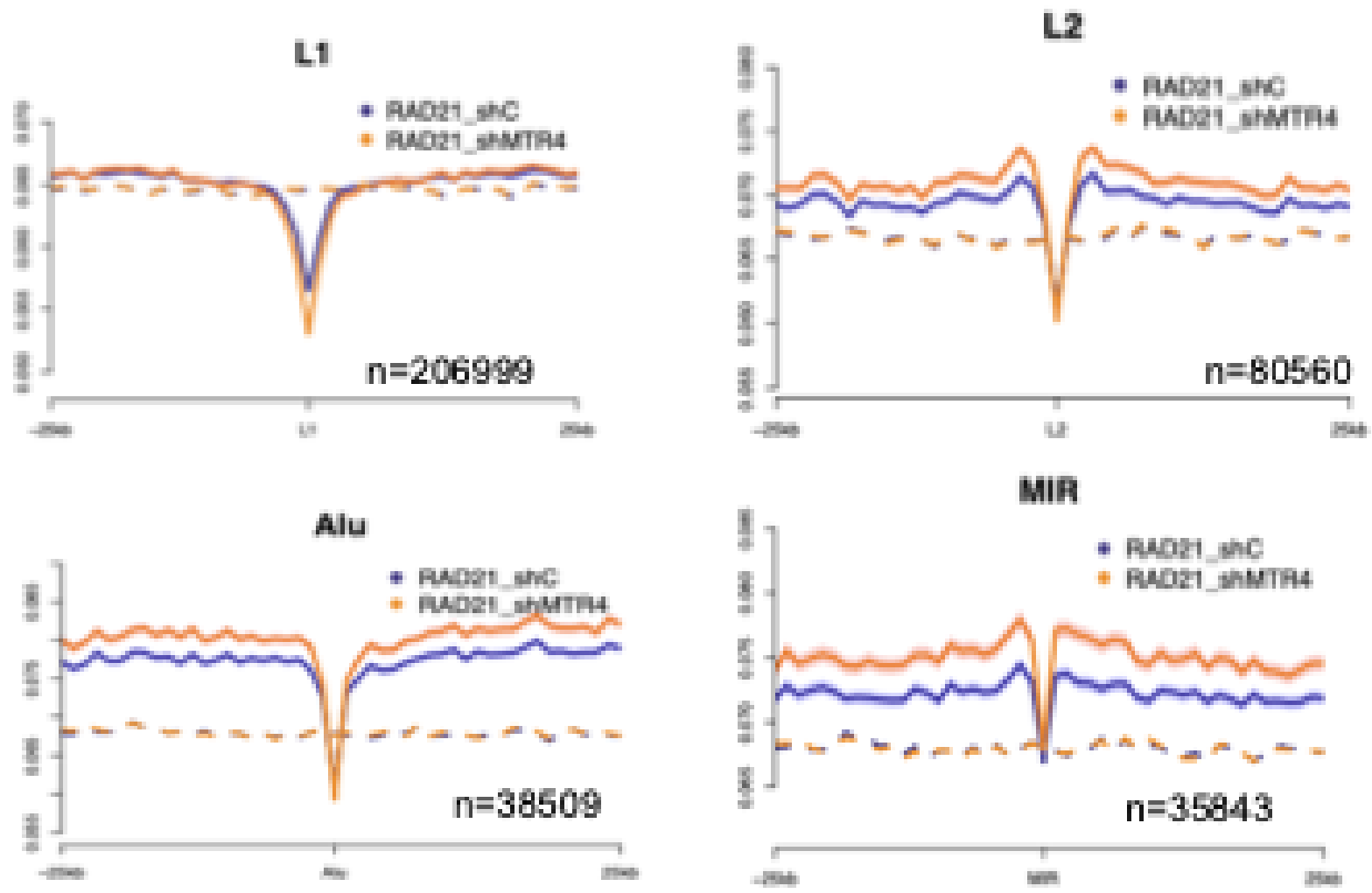

Rev Figure 1. Analysis of Rad21 ChIP-seq signal in control (shC) or MTR4-depleted (shMTR4) cells at LINE1 (L1), LINE2 (L2), Alu and MIR sites, +/- 25 kB. Dashed lines represent randomized positions of the same length and chromosome distribution as the target sites.

The overlap among the chromatin binding profiles of MTR4, ZCCHC8, and ZFC3H1 is minimal. Does this indicate functional diversity or moonlighting functions for these factors? If so, it is critical to conduct analyses using enzymatic mutants to clarify the specific roles of the nuclear exosome.

Response:

We thank the reviewer for raising this point. Minimal overlap is expected between ZFC3H1 and ZCCHC8, as they are components of distinct nuclear exosome targeting complexes, PAXT and NEXT, respectively. In contrast, given our previous work showing strong overlap among the genomic localization of several PAXT subunits (Contreras et al., 2023), it was surprising to observe rather limited overlap between MTR4 and the other factors. Despite the partial overlap, our RNA-seq analyses demonstrate that depletion of each factor stabilizes a highly overlapping set of PROMPTs. This functional convergence supports the interpretation that these cofactors largely act within the same nuclear exosome pathway. Furthermore, experiments added during revision (Supplementary Figure 6 D and E) show that the PAXT subunit, ZFC3H1, affects enhancer-promoter contacts similarly to MTR4. This suggests that the effects on enhancer-promoter contacts are likely due to nuclear exosome targeting complexes.

As the authors claim effects on enhancer-promoter interactions, they should provide evidence of specific loops formed between enhancers and promoters. Since Hi-C resolution is low, high-resolution 4C analysis would be advantageous. Additionally, snapshots of enhancer and promoter binding signals, as well as changes in specific PROMPTs, should be presented.

Response:

We appreciate the reviewer's suggestion regarding higher-resolution analyses of enhancer–promoter loops. While 4C would indeed provide locus-specific detail, our Hi-C datasets in HeLa cells offer

sufficient resolution to detect changes in enhancer–promoter interactions at the scale relevant to PROMPTs. To further strengthen this point, we have now included representative browser snapshots showing enhancer–promoter contacts together with the corresponding stabilization of PROMPTs (Figure 3b). Importantly, APA increases the effective resolution of Hi-C data to approximately 1 kb at inspected regions, as demonstrated both in our previous work and by Lieberman-Aiden's group (Liang et al. *Mol Cell* **53**, 672–681,2014; Rao et al. *Cell* **159**, 1665–1680, 2014).

What is the specificity of MTR4's effects on enhancer-promoter interactions? Can the authors analyze chromatin structure data post-knockdown by subgrouping based on MTR4 binding presence/absence and its effects on PROMPTs?

Response:

We thank the reviewer for this insightful suggestion. We have now stratified the post-knockdown Hi-C datasets (following MTR4 depletion) according to whether MTR4 binding sites are associated or not with MTR4-sensitive PROMPTs or eRNAs, respectively. This analysis reveals that regions producing MTR4-dependent PROMPTs or eRNAs are significantly more likely to display altered chromatin interactions upon depletion, supporting the view that PROMPT and eRNA stabilization contributes to the observed changes in 3D genome organization. The results of this analysis are presented in Supplementary Figure 6C and are described in the results section (lines 292-299).

Overall, the ncRNA-based analyses strengthen the functional link between MTR4-mediated ncRNA surveillance and alterations in chromatin architecture.

Additionally, given that the authors possess RNA-seq data, do the genes affected by PROMPTs exhibit changes in expression? Are these differentially expressed genes enriched in specific functional pathways?

Response:

We thank the reviewer for this suggestion. We performed gene ontology analyses on genes that were significantly upregulated or downregulated following MTR4 depletion. The enriched biological pathways identified from this analysis are presented below in Rev Figure 2. For upregulated genes, an FDR threshold of 0.1 was used, as applying a more stringent cutoff (FDR ≤ 0.05) did not yield any pathways reaching statistical significance.

At this stage, we have not incorporated these results into the main manuscript but would be happy to do so should the reviewer consider the inclusion of this analysis valuable.

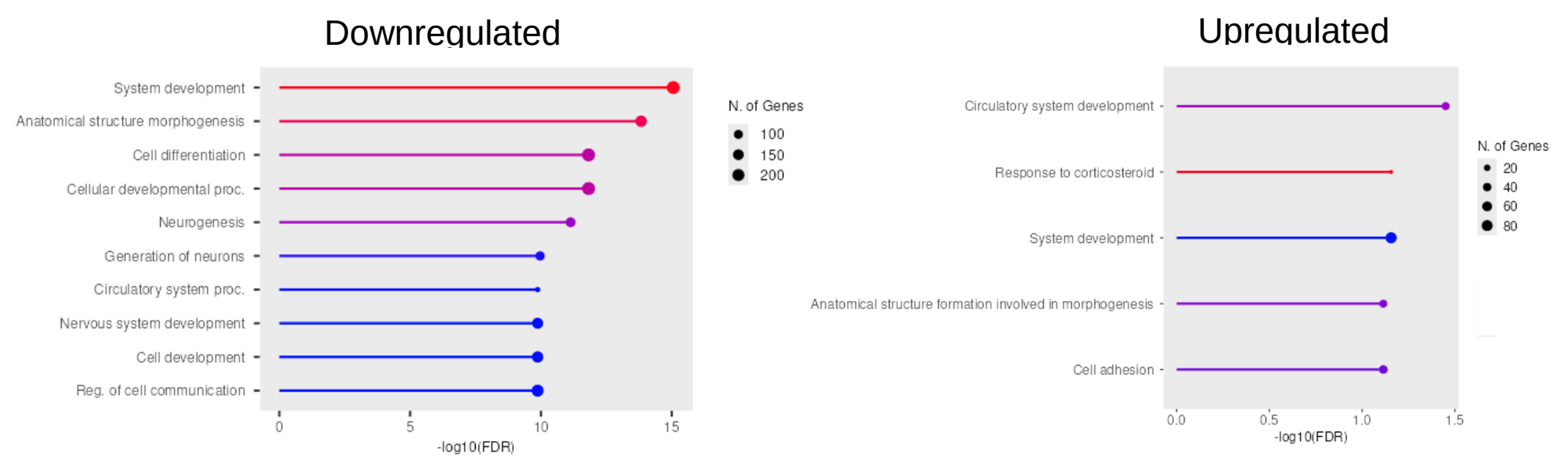

Rev. Figure 2. Gene ontology analysis showing biological pathways affected by genes that were either downregulated or upregulated, as indicated, following knock-down of MTR4. Colour scale represents -log10(FDR).

In Figure 4, the authors show that the knockdown of ZFC3H1 leads to an increase in RAD21. Does this knockdown also induce defects in 3D chromatin organization? Moreover, if ZFC3H1 influences PROMPTs at TSSs, how does it concurrently affect RAD21? Lastly, does MTR4 have any effect on enhancer RNAs?

Response:

We thank the reviewer for these insightful questions. To address them, we have now performed Hi-C following ZFC3H1 depletion. These data reveal chromatin interaction changes highly similar to those observed upon MTR4 knockdown (Supplementary Fig. 6D-E), supporting the conclusion that loss of nuclear exosome targeting activity broadly impacts 3D chromatin organization. We have clarified this in the Results and Discussion.

Regarding RAD21 enrichment, our new analyses indicate that ZFC3H1 depletion affects enhancer–promoter contacts in the same direction as MTR4 depletion. Because both proteins act within nuclear exosome targeting complexes, the most consistent interpretation is that their effects on cohesin arise from the shared consequence of ncRNA stabilization. Indeed, ZFC3H1-sensitive PROMPTs accumulate at TSSs and enhancers, and these are the same loci that exhibit increased RAD21 occupancy. Thus, the influence of ZFC3H1 on cohesin binding is most parsimoniously explained by enhanced ncRNA retention at regulatory elements, which promotes cohesin tethering and alters local chromatin architecture.

Finally, with respect to enhancer RNAs, we observe reproducible increases in eRNAs upon MTR4 depletion (Supplementary Fig. 2F), similar in proportion to PROMPT stabilization. New analysis also shows that enhancer-promoter LRIs increase significantly in MTR4-depleted cells at sites bound by MTR4 in control cells and that accumulate eRNAs, or PROMPTs upon MTR4 depletion (Supplementary Figure 6C).

Reviewer #2

This study comprehensively investigates the role of RNA Exosome targeting complexes in regulating three-dimensional (3D) chromatin structure. The findings reveal notable discrepancies in genome occupancy among NEXT, PAXT, and the RNA helicase MTR4, despite the helicase's established role in cooperating with both NEXT and PAXT. Furthermore, MTR4 knockdown via RNA interference (RNAi) resulted in increased cohesin levels, particularly an elevated Rad21 signal at chromatin loops. Based on these observations, the authors propose that the RNA helicase MTR4 regulates enhancer-promoter interactions by modulating the levels of non-coding RNAs (ncRNAs).

However, further clarification and additional experimental evidence are necessary to substantiate these claims. As MTR4 operates in concert with NEXT, PAXT, and the RNA Exosome complex, isolating the specific role of MTR4 from its interactions with these complexes is inherently challenging. To address this issue, it is essential to examine the effects of MTR4 knockdown in a context where NEXT or PAXT depletion does not interfere. For instance, identifying ncRNAs exclusively affected by MTR4 knockdown, but not by NEXT or PAXT depletion, would help delineate the MTR4-specific effects. In this context, relying solely on RNA-seq after prolonged (72-hour) depletion of each component may not provide adequate resolution to distinguish their specific targets. Instead, additional experimental approaches, such as nascent RNA analysis or CLIP-seq, would be more suitable for capturing real-time transcriptional activity and identifying the direct binding sites of these complexes.

Response:

We appreciate the reviewer's thoughtful comments and agree that disentangling the specific contribution of MTR4 from its association with NEXT, PAXT, and the RNA exosome is inherently challenging. Our intention was not to suggest that MTR4 functions entirely independently, but rather to illustrate how MTR4 activity within these complexes influences chromatin organization.

Consistent with this, RNA-seq analyses from this study and published studies (Lubas et al, 2011; Meola et al, 2016; Ogami et al, 2017) show that depletion of MTR4, ZFC3H1, or ZCCHC8 leads to stabilization of largely overlapping sets of PROMPTs, supporting the view that these cofactors act in a convergent pathway. We have revised the text to clarify this point and to avoid implying MTR4-exclusive roles.

We also agree that additional approaches, such as nascent RNA-seq or CLIP-seq, would be valuable for mapping direct binding sites and capturing the immediate transcriptional consequences of factor loss. While these experiments are beyond the scope of the present work, we wish to pursue these approaches, together with acute protein degration methods, in future work.

Finally, we have added new data showing that ZFC3H1 has a similar effect to MTR4 on enhancer-promoter contacts (Supplementary Figure 6D-E), suggesting that nuclear exosome targeting complexes, rather than MTR4 alone, are implicated in this function.

The following points are to be reconsidered:

⟩ *First, the authors have repeatedly used the terms "nuclear exosome targeting complexes" and "nuclear RNA exosome" interchangeably throughout the manuscript. However, these are distinct protein complexes with fundamentally different roles. It is recommended to use the term "nuclear exosome targeting complexes/helicase" specifically when referring to NEXT, PAXT, and MTR4 to avoid confusion and ensure terminological precision.*

Response:

We thank the reviewer for highlighting this important distinction. We have carefully revised the manuscript to ensure consistent terminology. Specifically, we now use "nuclear exosome targeting complexes/helicase" when referring to NEXT, PAXT, and MTR4, and reserve "nuclear RNA exosome" for the catalytic degradation complex.

There are several typographical errors and figure number misrepresentations throughout the manuscript that need to be addressed for clarity and accuracy:

⟩ *Line 55: Replace "(TADS)" with the correct term "TADs."*

⟩ *Line 215: Replace "Aggregated plot analysis" with the term "Aggregated peak analysis"*

⟩ *Line 251: Update "Fig. 5c" to "Fig. 4c."*

⟩ *Line 273: Correct "Supplementary Fig. 6A" to "Fig. 5a."*

⟩ *Line 294: Update "Fig.5D" to "Fig.5d"*

Response:

We appreciate the reviewer's careful reading of the manuscript and for pointing out these typographical and figure reference errors. We have corrected all of the noted issues, including "TADS" to "TADs," "Aggregated plot analysis" to "Aggregated peak analysis," and the mislabelled figure references. We have also re-checked the manuscript systematically to ensure that figure citations and terminology are now consistent throughout.

Add references to following descriptions are recommended

ν *Line62, Line67*

Response:

We thank the reviewer for this helpful suggestion. We have now added the appropriate references at lines 62 and 67 (lines 64 and 69, revised manuscript) to support the statements.

In Figure 5b, to avoid potential confusion, it is recommended to use the term "chromatin loop" (or "EP loop") instead of "Central area." This terminology would provide greater clarity and align with commonly accepted nomenclature in the field.

Response:

We appreciate this suggestion. To avoid confusion and align with standard terminology, we have updated the label "Central area" to "E-P loop" in Figure 5b. The figure legend has also been revised accordingly.

⟩ In Figure 5d, although the figure represents the condition of MTR4 depletion, the presence of MTR4 is still depicted. Additionally, the transcription of PROMPTs appears to increase, as indicated by the red arrow. Is there any evidence supporting the claim that MTR4 knockdown (KD) leads to increased transcription of PROMPTs? If the primary effect is ncRNA accumulation rather than enhanced transcription, the figure should depict a red arrow of the same size accompanied by an increased quantity of RNAs. Finally, to accurately reflect the observed accumulation of Rad21, it would be more appropriate to illustrate an increased number of cohesin rings in the MTR4-KD condition.

Response:

We thank the reviewer for this careful observation. Figure 5d has been revised accordingly: MTR4 protein is now shown as depleted, transcription arrows are kept constant while the amount of RNA is increased to reflect accumulation rather than enhanced transcription, and additional cohesin rings have been added to represent the observed increase in Rad21 binding. These changes provide a more accurate and clearer representation of our data.

⟩ In the discussion, the proposed model suggests that accumulated ncRNAs may stabilize cohesin loops. However, have you also considered the possibility that the observed increase in Rad21 at these regions could be attributed not to ncRNAs, but rather to changes in chromatin accessibility or region availability following MTR4 depletion? Exploring this alternative explanation could provide additional insights into the underlying mechanisms.

Response:

We agree with the reviewer that increased Rad21 occupancy could alternatively reflect changes in chromatin accessibility or regional availability following MTR4 depletion. We have now incorporated this as an additional plausible mechanism in the Discussion (lines 340-349).

⟩ Further Recommendations for Figure Representations:

1. Resolution Issues: Several figures have low resolution, making them appear blurred. Improving the resolution of these figures is essential to ensure clarity in data presentation.

Response:

We thank the reviewer for pointing this out. We have regenerated the figures with improve clarity and ensure that all data are clearly presented.

2. Figure 1(b): The representation of p-values should be corrected for consistency and proper formatting. For instance, change “1e-126” to “1 × 10^-126” or “1e-126.”

Response:

We thank the reviewer for this suggestion. The representation of p-values has been standardized for consistency and clarity, using the format ‘$p < 1 \times 10^{-126}$’ throughout.

3.Figure 1(c-e): Include an explanation for the p-value representation (***) in the figure legend or directly within the figure. This clarification is necessary for readers to interpret the significance levels accurately.

Response:

We appreciate the reviewer's suggestion. We have added an explanation of the significance symbols (*****) in the legend for Figure 1c–e, so that readers can clearly interpret the p-value thresholds used in these panels.

4. Figure 2(b): In the left-bottom Venn diagram, the sum of MTR4 peaks is reported as 5672, while in other figures, the sum is 5666. Please clarify the discrepancy or provide an explanation for the difference.

Response:

We thank the reviewer for catching this discrepancy. We have corrected the figure and ensured that the numbers are consistent throughout the manuscript.

5. Figure 5(c): Represent the size of the Hi-C matrix in base pairs (bp) to provide a more comprehensive understanding of the data resolution. Adding this detail will enhance the interpretability of the figure.

Response:

We thank the reviewer for this helpful suggestion. We have updated Figure 5c to include the size of the Hi-C matrix in base pairs.

I strongly believe addressing these points would enhance this study's comprehensibility, accuracy, and validity.

Response:

We thank the reviewer for this comprehensive and constructive feedback. We have carefully addressed the points raised and believe that these changes have improved the clarity, accuracy, and overall quality of the manuscript.

Reviewer #3

In their manuscript "Nuclear exosome targeting complexes modulate cohesin binding and enhancer-promoter interactions in 3D", Akkawi et al. implicate exosome targeting complexes in the regulation of 3D genome contacts. This is supported by data demonstrating the localization of exosome targeting factors to DNA contact points (in particular enhancers and TSS) and chromatin conformation capture experiments comparing targeting factor knockdown and controls. They further provide evidence that the changes in genome conformation are reflected in altered cohesion levels at the contact sites where exosome targeting factors are present.

This is an interesting, well-conducted study highlighting the responsiveness of the genome to the non-coding transcriptome and the role of nuclear RNA surveillance factors in shaping this landscape. Consider correlating eRNA stabilization with chromatin contacts.

Response:
We are grateful to the reviewer for their thoughtful and encouraging assessment of our study. We are pleased that they found the work well conducted and that our findings provide meaningful insight into how nuclear RNA surveillance factors shape the interplay between non-coding RNAs and 3D genome organization.

Regarding the suggestion to correlate eRNA stabilization with chromatin contacts, we agree that this is an important point. We have therefore extended our analysis to assess whether stabilization of eRNAs or PROMPTs upon MTR4 depletion, together with MTR4 occupancy, correlates with changes in long-range interactions (LRIs). These new results, now presented in Supplementary Figure 6C and described in the Results section (lines 292–299), show that the strongest LRI changes in MTR4-depleted cells occur at sites that are bound by MTR4 in control cells, and that accumulate a ncRNA (eRNA or PROMPT) upon loss of MTR4. Collectively, these findings further support our model that stabilized ncRNAs contribute to the observed changes in chromatin architecture following MTR4 depletion.

General comments:

The figures are rich in information, and additional details in the figure legends would sometimes aid in interpreting the data (see below).
In the discussion, the authors state: "It seems unlikely that MTR4 or associated complexes, such as PAXT, are directly responsible for the effects observed on LRIs. Instead, it seems more probable that ncRNA targets of nuclear exosome, particularly eRNAs and PROMPTs, that were concomitantly stabilized at the enhancers and promoters in contact, may be responsible for the effect on LRIs and loop extrusion.". This s a very reasonable assumption. But - since the authors have both RNA expression and chromosome conformation data available for control and targeting factor knockdown – why not use

the available data to explore correlations that may directly support that idea? This was done for PROMPTs to some extent, but not for eRNA.

Response:

We thank the reviewer for the suggestion. As noted above, we have extended our analyses to assess the relationship between eRNA and PROMPT stabilization and long-range interactions (LRIs). The new analyses show that loci bound by MTR4 in control cells, which accumulate eRNAs or PROMPTs upon MTR4 depletion, are significantly more likely to exhibit increased enhancer–promoter contacts in MTR4-depleted cells. These findings support our model that stabilization of non-coding RNAs contributes to 3D genome remodeling. These results are now presented in Supplementary Figure 6C and are described in the Results section (lines 292-299).

Minor points:

Figure 2a shows the distribution of ChIP-seq peaks across different features, including TSS, TES, gene bodies and intergenic. Later in the manuscript, the main focus is on TSS and enhancers. I assume enhancers are part of the intergenic features. Would it be possible to include the information in this figure, e.g. as percentage of intergenic peaks that correspond to enhancers (and maybe also percentage of TSS that are associated with a PROMPT)? It might also be good to include absolute numbers for the counted peaks in each category. Alternatively, a metagene analysis as in Fig 1c-e could be repeated split into peaks associated with the different features?

Response:

We thank the reviewer for this valuable suggestion. We have updated Figure 2a to indicate the percentage of intergenic peaks that correspond are marked with H3K4me1, a mark of enhancers. We have also included absolute numbers for each category.

Figure 2b: It would be interesting to know whether the overlap of ChIP peaks between samples is determined by the features that the peaks are found at. Would it be possible to integrate that information in figure 2b, e.g. by including miniatures of bar plots similar to the ones shown in 2a?

Response:

We thank the reviewer for this suggestion. We have included this analysis in Supplemntary Figure 2G.

I am confused about the labelling of some bubbles in Fig 2b, and Supp. Fig 3 (all panels) as "PROMPTs DETECTED in cells depleted…". What is the cut-off for detection? Why not show the ones that are differentially expressed upon knock-down (and state the cut-off for that)?

Response:

We thank the reviewer for highlighting this point of confusion. The bubbles in Figure 2b and Supplementary Figure 3 represent numbers of differentially expressed PROMPTs or genes upon knockdown of ZFC3H1, ZCCHC8 or MTR4. We have clarified this in the figure legends and Methods, and we now specify the statistical threshold used for calling differential expression (FDR < 0.05).

Line 176/177: "In contrast, MTR4 recruitment sites were enriched in the histone H3K4me1 mark that is associated with enhancers" – To me, this sound as if Z3 and Z8 were not enriched at enhancers, but they are (even more strongly than MTR4 according to Supp Fig 2G). Please change the wording accordingly.

Response:

We thank the reviewer for pointing out the confusing wording. The text has been revised (lines 181-183) to indicate that all three factors are enriched at enhancers.

Abstract: "While nuclear RNA exosome, … control" ◊ "While the nuclear RNA exosome, … controls"

Response: We corrected the sentence for grammatical accuracy.

Line 162/163: "previous analyses showing the contribution of MTR4-containing complexes in regulating ncRNAs including eRNAs and PROMPTs" ◊ please include references

Response: We have added references to support the statement regarding the contribution of MTR4-containing complexes in regulating ncRNAs, including eRNAs and PROMPTs (line 168).

In Supp. Fig 3A, B, and C, the numbers of genes do not fully add up (B + C is not equal to A).

Response: We thank the reviewer for pointing out the discrepancy in gene numbers. The figures have been corrected so that the counts now add up consistently across panels.

Line 203/204: "LRIs with distant TSSs that accumulate PROMPTs upon nuclear exosome depletion" – To me, the wording suggests that the PROMPTs are retained on the TSS at the chromatin. Please change in a way that it is clear you refer to RNA levels.

Response: We have revised the wording (lines 216-218) to clarify that the reference is to RNA levels rather than retention at the TSS, improving clarity for the reader.

Lines 281, 286, 291, 292 and others: "nuclear RNA exosome complexes" ◊ "nuclear RNA exosome targeting complexes"

Response: We have standardized terminology throughout the manuscript, replacing "nuclear RNA exosome complexes" with "nuclear RNA exosome targeting complexes" where appropriate.

Lines 406-408: "Preliminary analyses were performed in comparison with…". ◊ Was this included in the manuscript? If not, remove?

The preliminary analyses referenced were not included in the manuscript; we have removed this sentence.

Figure 3b: To make the browser shots easier to interpret, it would be helpful if relevant features (TSS, enhancers) could be marked. Potentially combine with browser shots for RNAseq data (siMTR4, siZ3) for the location of PROMPTs?

Response: We thank the reviewer for this helpful suggestion. We have updated Figure 3b to clearly mark relevant genomic features, including histone marks associated with enhancers (H3k4me1 and H3K27ac), and have shown the location of PROMPTs.

Figure 3c: Please specify how "enrichment in PROMPTs" was determined. Is this the log2FC for reads in siMTR4 vs control? Does this graph include LRI scores for all enhancer-TSS contacts genome-wide (in that case, I would expect at least the three lower quartiles to reflect zero interactions), or was this analysis performed on a window as stated for Figure 4a ("all active TSSs within 1Mbp")? Please specify. Out of interest, could you also include summary stats for LRIs in the different quartiles for MTR4-bound and non-bound enhancers (in the supplements?). This could reveal a general trend of the impact of the presence of MTR4 on LRIs.

Response: We thank the reviewer for these detailed comments. The "enrichment in PROMPTs" in Figure 3c is calculated as the $\log_2$ fold change of reads in siMTR4 versus siControl. The analysis was performed on all active TSSs within a 1 Mbp window around enhancers, consistent with the approach described for Figure 4a. This information has been added to the legend of Figure 3c.

Figure 3d: Scale in grayscale, figure uses a coloured scale. Arrows almost point to the centre of the plot, but not quite. Is that intended?

Response: We thank the reviewer for pointing this out. The figure has been updated to include a correctly labelled color scale. We also adjusted the arrows so that they accurately indicate the intended positions on the plot. Please note that the figure is now Supplementary figure 3G.

Fig 5a & Suppl. 4C: Please include scale.

Response: We thank the reviewer for pointing out this error. The issue has been corrected in the revised manuscript.

REVIEWERS' COMMENTS

Reviewer #1:

The authors have primarily addressed my comments by expanding the discussion, with limited new experimental data. If the authors agree that the novelty of the manuscript and the potential for indirect effects remain as concerns, I recommend performing acute protein degradation experiments and related RNA-seq analyses to better exclude indirect effects.

On one hand, the authors have not provided high-resolution data on E–P conformational changes; on the other, they have not clarified how ZFC3H1 regulates RAD21. Thus, the current evidence supporting the extension of the conclusion to Cohesin binding and E–P interactions is insufficient for publication in Nature Communications.

Response to Reviewer #1

We thank the reviewer for their careful evaluation of our revised manuscript and for acknowledging the additional discussion provided. We appreciate the concerns raised regarding the extent of new experimental data and the possibility of indirect effects.

While we agree that acute protein degradation approaches could provide valuable complementary insights, such experiments are beyond the scope of the present study. Instead, we have strengthened the manuscript by integrating multiple orthogonal datasets, including chromatin immunoprecipitation, RNA sequencing, and chromatin conformation capture analyses. Together, these approaches consistently support a model in which nuclear RNA exosome targeting factors influence cohesin occupancy and enhancer–promoter interactions in association with the accumulation of noncoding RNAs.

Regarding the resolution of enhancer-promoter conformational changes, we note that our conclusions are based on genome-wide chromatin conformation capture analyses, which are well established for assessing changes in long-range interactions and loop architecture at the scale investigated here. While higher-resolution approaches may further refine these observations, the current data provide robust evidence for alterations in both anchor contacts and intra-loop interactions.

With respect to the mechanism by which ZFC3H1 influences RAD21 occupancy, we agree that this remains an important open question. We have clarified in the revised manuscript that

our data support a functional association rather than a direct mechanistic link, and we have discussed potential indirect effects mediated by the accumulation of noncoding RNAs.

Overall, while we acknowledge the limitations noted by the reviewer, we believe that the combined evidence presented provides a coherent and reproducible framework supporting the interplay between nuclear RNA exosome activity, noncoding RNA accumulation, and cohesin-mediated genome organization.

Reviewer #2:

I have carefully reviewed the authors' revised manuscript and the point-by-point response to the reviewers. The authors have provided clear experimental evidence, expanded explanations, and appropriate revisions throughout the manuscript, which collectively strengthen the scientific rigor and clarity of the work. Therefore, I recommend the manuscript for acceptance in its current form.

Response:

We thank the reviewer for their careful evaluation of the revised manuscript and for their positive assessment. We are grateful for their recognition of the improvements in clarity and scientific rigor, and we appreciate their support for publication.

Reviewer #3:

All points that I have raised have been satisfactorily addressed in the revision.

Response:

We thank the reviewer for their thoughtful comments on the initial submission and for acknowledging that their concerns have been satisfactorily addressed in the revised manuscript. We appreciate their positive evaluation.
